# Supramolecular peptide nano-assemblies for cancer diagnosis and therapy: from molecular design to material synthesis and function-specific applications

**DOI:** 10.1186/s12951-021-00999-x

**Published:** 2021-08-23

**Authors:** Yan Wang, Xiaoyuan Zhang, Keming Wan, Nan Zhou, Gang Wei, Zhiqiang Su

**Affiliations:** 1 College of Chemistry and Chemical Engineering, Qingdao University, 266071 Qingdao, People’s Republic of China; 2 State Key Laboratory of Chemical Resource Engineering, Beijing Key Laboratory of Advanced Functional Polymer Composites, Beijing University of Chemical Technology, Beijing, People’s Republic of China

**Keywords:** Peptide self-assembly, Hybrid nanomaterials, Cancer, Diagnosis, Therapy

## Abstract

Peptide molecule has high bioactivity, good biocompatibility, and excellent biodegradability. In addition, it has adjustable amino acid structure and sequence, which can be flexible designed and tailored to form supramolecular nano-assemblies with specific biomimicking, recognition, and targeting properties via molecular self-assembly. These unique properties of peptide nano-assemblies made it possible for utilizing them for biomedical and tissue engineering applications. In this review, we summarize recent progress on the motif design, self-assembly synthesis, and functional tailoring of peptide nano-assemblies for both cancer diagnosis and therapy. For this aim, firstly we demonstrate the methodologies on the synthesis of various functional pure and hybrid peptide nano-assemblies, by which the structural and functional tailoring of peptide nano-assemblies are introduced and discussed in detail. Secondly, we present the applications of peptide nano-assemblies for cancer diagnosis applications, including optical and magnetic imaging as well as biosensing of cancer cells. Thirdly, the design of peptide nano-assemblies for enzyme-mediated killing, chemo-therapy, photothermal therapy, and multi-therapy of cancer cells are introduced. Finally, the challenges and perspectives in this promising topic are discussed. This work will be useful for readers to understand the methodologies on peptide design and functional tailoring for highly effective, specific, and targeted diagnosis and therapy of cancers, and at the same time it will promote the development of cancer diagnosis and therapy by linking those knowledges in biological science, nanotechnology, biomedicine, tissue engineering, and analytical science.

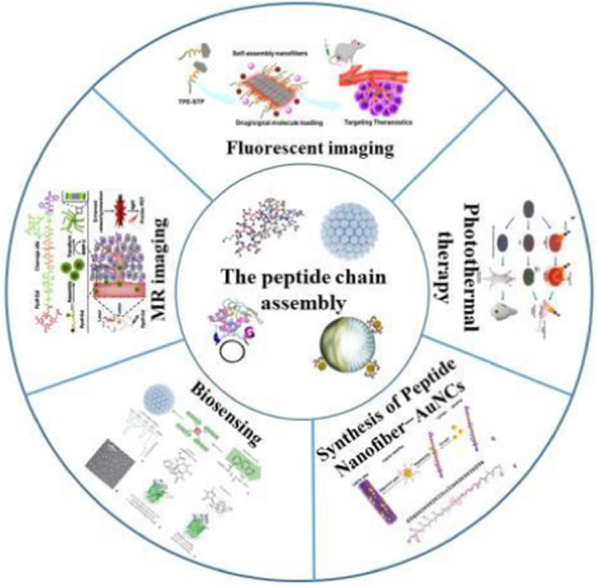

## Introduction

Cancer is one of the major diseases that seriously threaten human life currently due to the effects of environmental pollution, tobacco use, and other unhealthy lifestyles.. According to the World Health Organization, there were about 18 million new cancer cases in 2019, which caused millions of deaths worldwide and trillions of US dollars economic loss each year. Therefore, the early diagnosis and clinical therapy of cancers is one of the current research directions that the international community pays attention to [1].The diagnosis of cancers could be achieved by some traditional and advanced techniques, such as traditional bioanalysis of carcer biomarkers, biosensors, optical imaging, nuclear magnetic resonance imaging, and others. Nanomaterial-based diagnosis exhibited great advantages due to their low cost, quick detection, simple analysis, and high sensitivity [2]. In addition, traditional clinical therapy of cancers includes surgery, radiotherapy, and chemotherapy. However, these therapy methods also have some shortcomings. For example, the surgery and chemotherapy will bring physical pain to the patient, and will be accompanied by a higher recurrence rate; while the radiotherapy and chemotherapy will kill normal cells and reduce the body's immunity at the same time when killing cancer cells [3, 4]. Thanks to the quick development of nanotechnology and biological science, most of the problems mentioned above could be solved effectively by applying functional nanomaterials and novel nanobiotechniques, revealing huge potential prospects of nanotech in the biomedical community [5]**.**

Previous studies have indicated that nanomaterials showed great applications for the diagnosis and therapy of cancers. Various nanomaterials, including noble metal nanomaterials (such as gold, silver nanoparticles, nanorods, nanowires, etc.) [6, 7], semiconductor nanoparticles [8, 9], carbon materials [10, 11], two-dimensional (2D) materials [12, 13], and polymer nanoparticles [14, 15] have been widely utilized for the detection and therapy of different cancers. Although these functional nanomaterials revealed high performance on both diagnosis and therapy of cancers, their low biocompatibility, unknown long-term safety, and low biodegradability limited their wide applications in practical clinical therapy. To overcome these problems, it is highly necessary to develop the design and synthesis of functional nanomaterials with high biocompatibility, specific targeting, and good biodegradability.

Several potential solutions could be applied in response to the above problems. Firstly, the biocompatibility of nanomaterials can be improved by polymer modification [16–18]. For instance, the modification of graphene [16] and WS_2_ [17] nanosheets with polymers could significantly improve the physiological stability and biocompatibility of materials. Secondly, in order to improve the biological targeting properties of the material, the surface of the material can be modified with molecules that specifically interact with tumor cells [19–21]. Zhang et al*.* reported the modification of MoS_2_/Cu_1.8_S with ssDNA aptamer and polyethylene glycol (PEG) for multifunctional bioimaging, chemical, and photothermal therapy of cancers [20]. It was found that the introduction of aptamer into the nanomaterial system significantly enhanced the target recognition ability of the material and promoted the precise releasing of anti-cancer drugs. Finally, in order to promote the biodegradability of nanomaterials, various biomolecules, such as proteins [22–24], DNA [25, 26], and peptide [27–29] have been used for the biomimetic synthesis and modification of nanomaterials for controlled cancer diagnosis and therapy.

Peptide-based nanomaterials have shown wide applications in the fields of biomedicine, tissue engineering, biosensors, nanodevices, energy storage, environmental science, and many others due to their high biocompatibility, good degradability, controlled self-assembly, and unique biomimetic properties [30–32]. In addition, the use of tailorable design of the amino acid sequence of the peptide molecules can effectively regulate the structure and function of peptide-based self-assembled nanomaterials [33], which provided great supports on the diagnosis and therapy of cancers in a certain extent. Previously, a lot of reviews on the design, synthesis, self-assembly, and applications of peptide-based nanomaterials have been released. For instance, Yan and co-workers summarized recent advance in the design of nanodrugs based on peptide self-assembly for drug delivery and tumor therapy applications [34], Guo et al*.* presented recent progress of the synthesis and cancer treatment of therapeutic peptide-based nanomaterials [35], and Zhang et al*.* provided a review on the structural design, synthesis, types, and applications of peptide-based cancer vaccines, in which the roles of peptides on in regulating the immune systems for cancer treatment were discussed [36]. Although great achievement has been made in the last years, it is necessary for us to contribute a work to promote the understanding on the importance of peptide-based nano-assemblies on the diagnosis and therapy of cancers.

Herein, in this review, we present a comprehensive review on the design and synthesis of peptide-based nano-assemblies for the applications in cancer diagnosis and therapy. For this aim, in the next part (Part 2), we introduce the synthesis of various peptide nano-assembles, such as nanoparticles, nanospheres, nanofibers, nanotubes, nanosheets, nanobelts, vesicles, and hydrogels in detail. In addition, the formation of peptide-based hybrid nano-assemblies by combining peptide with other peptides, polymers, as well as zero-dimensional (0D), one-dimensional (1D), and 2D nanomaterials is also demonstrated and discussed. In Part 3, we summarize the advances in the design and creation of peptide-based nano-assemblies for cancer diagnosis, for example, in fluorescent imaging, magnetic resonance imaging, photoacoustic imaging, and biosensing of cancer cells. In Part 4, we provide the case studies on how to apply peptide-based nano-assemblies for cancer therapy, in which the therapy methods like peptide-mediated killing, chemo-therapy, photothermal therapy, and multi-therapy of cancer cells are introduced and discussed. In the final part, we point out the challenges remained currently and give our viewpoints of the potential efforts that could be done to enhance this promising research topic. We believe this review will contribute to bridge the studies on nanotechnology and biological science, promoting the developments of supramolecular nanomaterials in biomaterials science, biomedicine, tissue engineering, biosensors, and other corresponding aspects.

## Design and synthesis of peptide assemblies

In this part, the motif design, synthesis, and functional tailoring of pure peptide assemblies and hybrid assemblies are presented.

### Self-assembled peptide assemblies

Peptides play important roles in biomedical applications, which can act as signal molecules and hormones that are self-assembled into various forms of nanostructures, including 0D nanoparticles and nanospheres, 1D nanowires, nanotubes and spiral bundles, 2D nanosheets and nanoribbons, as well as three-dimensional (3D) vesicles and hydrogels. These peptide nanostructures have been widely applied for cancer diagnosis and therapy [37].

#### 0D peptide nanoparticles and nanospheres

Peptides are functional biomolecules that play key roles in life sciences, such as hormones and signaling molecules. It can self-assemble into 0D nanoparticles and nanospheres. The driving force in the process of self-assembly into advanced peptide nano-assemblies involves these internal and external molecular interactions like hydrogen bonding, hydrophobic, electrostatic interactions, π-π interaction, and others.

**Fig. 1:**
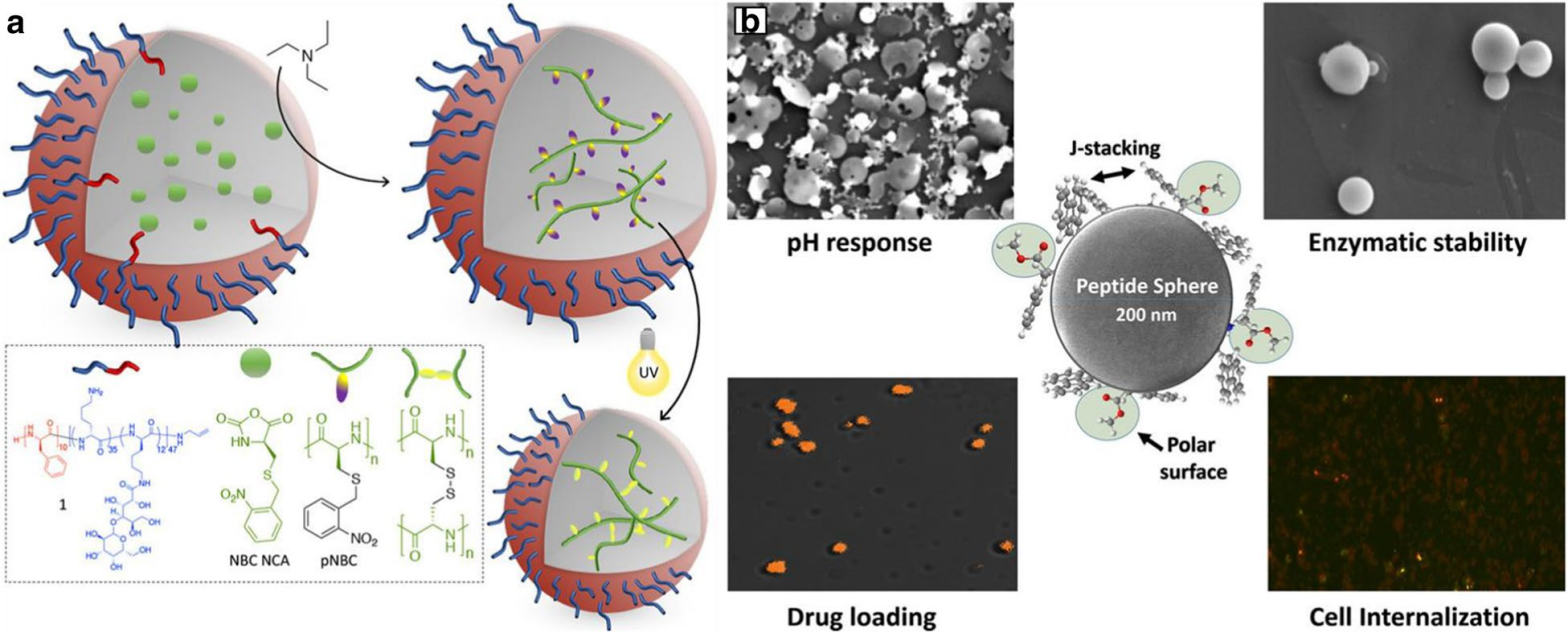
Design and synthesis of 0D peptide nano-assemblies: **a** Miniemulsion polymerization of NBC NCA and subsequent UV-cross-linking. Reproduced with permission from Ref. [42], Copyright 2019, American Chemical Society. **b** SPNS acts as single component, multifunctional drug delivery vehicle by addressing critical issues such as effective drug loading, pH responsive controlled release, biocompatibility, and stability against enzymatic degradation. Reproduced with permission from Ref. [45], Copyright 2020, Elsevier Ltd

Peptide structures with high biocompatibility can be used as biological nanoparticles for biotherapeutic platforms. Recently, it has been reported that synthetic peptides can be produced by the open-loop polymerization (ROP) of N-carboxylic anhydride (NCA), which can be used as an alternative method to traditional therapy [38, 39]. For example, star polylysine (L-lysine), with improved siRNA and pDNA loading, was reported to enhance the cargo protection ability and transfection properties. They can be synthesized by polymer-based treatment with drug-conjugated peptides [40, 41]. A facile method to synthesize peptide nanoparticles is to conduct emulsion polymerization in water by using monomers, initiators, and surfactant molecules. This technique has been widely used in industry to produce polymer emulsions. Nanoparticles synthesized through free radical polymerization can reach several tons. Using this method, Jacobs et al*.* demonstrated the preparation of polypeptide nanoparticles by monomer emulsion polymerization of amino acid NCAs (Fig. 1a) [42]. The S-(adjacent nitro benzyl)-(NBC) NCA 1 L-cysteine, sensitive to UV light, was selected as monomer for the synthesis of peptide nanoparticles. To achieve the aggregation of particles after cross-linking, they used a block of glycosylation peptide, which contained a poly(L-phenylalanine) block and a poly(L-lysine) block. This was a part of glycosylation lactam acid, amino acid, and macromolecular surfactant. They tried to take advantage of the tertiary amine NCA to promote the rapid activation of monomer (AMM) open-loop mechanism through the appropriate reaction conditions. Tumor-specific substances can design stimulus reactive polymer nanoparticles. It can lead them within the tumor programmed decomposition, thus releasing more easily spread in the tumors of low molecular weight components.

Peptide nanospheres are 0D polypeptide nanostructures that play an important role in medical fields such as drug and gene delivery. The π-π stacking and hydrophobic interaction between peptide molecules are of great importance on the formation of nanospheres. For example, A diphenylalanine (FF) peptide was a derivative of diphenylalanine with similar molecular properties to diphenylalanine peptide, which can self-assemble into nanospheres because their molecular structure is more rigid than 1D nanostructures with lower degrees of freedom. As they do not rotate around additional C–C bonds, and the steric hindrance is also higher. Similarly, the cysteine diphenylalanine tripeptide could form fullerene-like nanospheres via self-assembly [43].

Another type of nanosphere by the self-assembly of CG3R6TAT cationic peptide has been reported previously [44]. CG3R6TAT was modified with cholesterol as a hydrophobic tail. A fragment of CG3R6TAT, TAT, was used to construct antibacterial drugs. CG3R6TAT was composed of 3 glycine (G) as spacers and 6 arginine (R) residues, which enhanced the performance of the membrane translocation sequence, TAT. The designed peptide formed a core–shell micelle with cholesterol in the hydrophobic core, and with the cationic peptide outside the hydrophilic shell. Such core–shell micelles have a higher cationic charge density outside the cladding.

Currently, effective delivery of therapeutic drugs faces many challenges such as uncontrollable drug release, low drug bioavailability, degradation of the drug in the intestinal delivery process, and non-biocompatibility of the delivery carrier. To overcome these problems, Prabhjot et al*.* reported a single component based aqueous drug delivery system by generating self-assembled dipeptide nanospheres (SPNS) using Fmoc-L-Trp-L-Phe-OCH_3_ framework [45], as shown in Fig. 1b. The effects of hydrophobicity between dipeptides and π-π stacking in aqueous medium on the self-assembly process of peptide nanospheres were found. Initially, Fmoc-L-Trp-L-Phe-OCH_3_ was observed to constitute a spherical form of dipeptide in acetone solvent. Then, the stability of the spherical peptide nano-assemblies was optimized by using a 1:1 water/acetone binary solvent system, solving the problem of solvent applicability under physiological conditions. Slight turbidity in the addition of water indicates the formation of colloidal particles in the optimized binary solvent system. Interestingly, it has been observed that peptide molecules self-assemble into clearer spheres with an average diameter of 100–400 nm. After the above process, self-assembled peptide nanospheres (SPNS) were successfully dispersed in water, resulting in a biocompatible system suitable for drug delivery applications. Subsequently, the hydrophobic and hydrophilic domains of SPNS were examined. It was found that pH triggers changes in the morphology of SPNS and leads to the release of therapeutic drugs. Importantly, SPNS shows excellent stability to enzymatic degradation using chymotrypsin and low cytotoxicity to the HeLa cell line. Further, in vitro cell internalization and activity of released therapeutics from SPNS were analyzed against Escherichia coli (E. coli) (using ampicillin) and HeLa cell line (using doxorubicin). Notably, SPNS based drug delivery system observed to be highly stable against specific protease activity, which may help to regulate the concentration spikes of drugs due to uncontrolled release as well as increase the bioavailability of peptide-based therapeutics via oral administration. As a single-component multifunctional drug delivery carrier, the created SPNS was verified to be capable of solving key issues such as effective drug loading, pH-responsive controlled release, biocompatibility, and stability against enzymatic degradation.

#### 1D peptide nanofibers and nanotubes

**Fig. 2:**
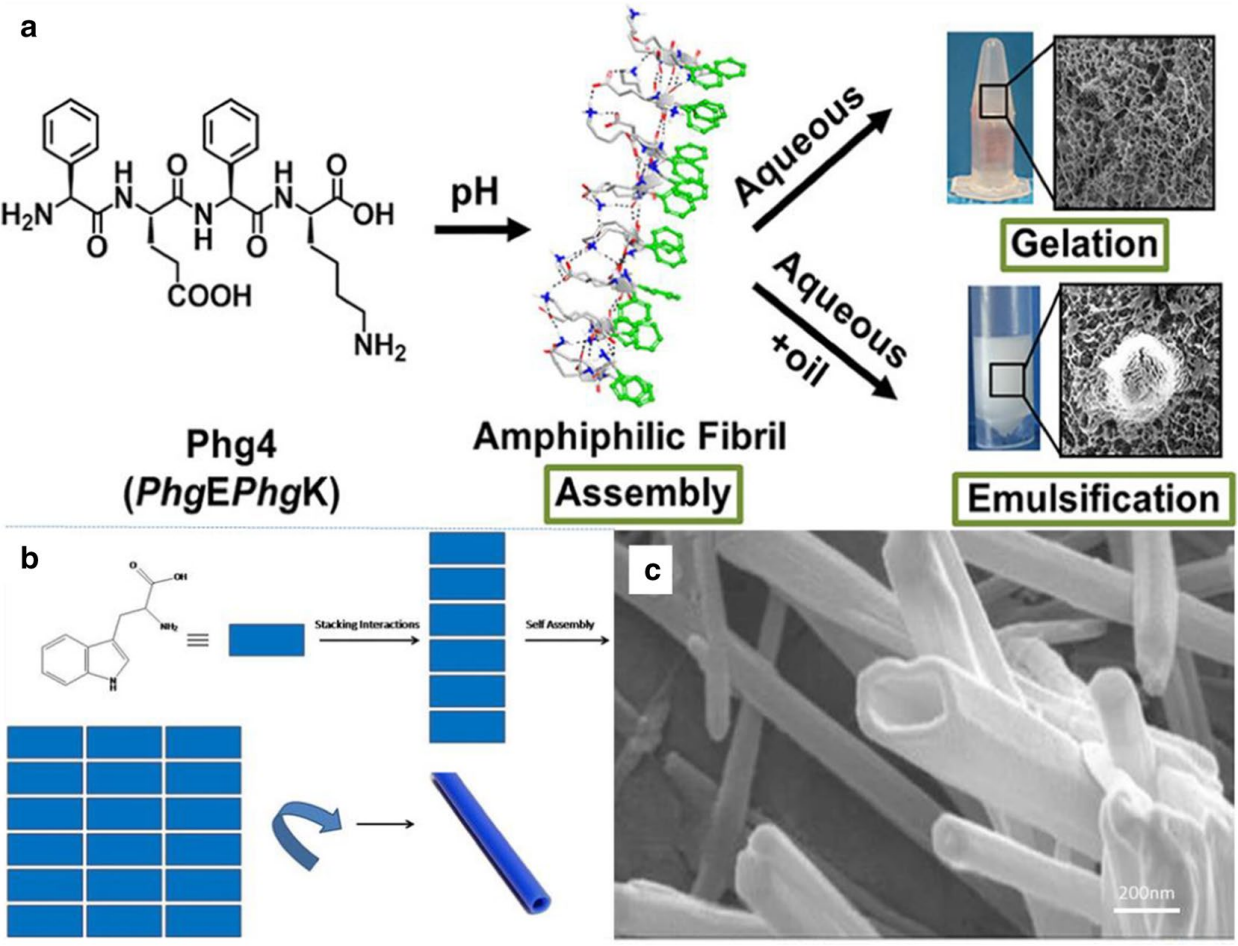
Design and synthesis of 1D peptide nanofibers and nanotubes: **a** Phg4 peptide self-assembled into a schematic diagram of nanofiber network. Reprinted with permission from Ref. [46], Copyright 2020, American Chemical Society. **b** The formation of nanotubes from amino acids precursors. **c** High-resolution SEM image of tryptophan nanotubes. Reprinted with permission from Ref. [52], Copyright 2017, Springer

Understanding the mechanism of peptide self-assembly and the stability of formed nano-assemblied is essential for the development of functional nanomaterials. Recently, Wychowaniec et al*.* adopted a rational design approach to demonstrate how a minimal structural modification to a non-assembling ultrashort ionic self-complementary tetrapeptide FEFK (Phe4) enhanced the stability of self-assembly into β-sheet nanofibers [46]. This specific self-assembly was achieved by replacing flexible phenylalanine (F) residue with the rigid phenylglycine (Phg), resulting in a constrained analogue (Phg)E(Phg)K (Phg4) which positioned aromatic rings in an orientation favorable for aromatic stacking. The designed Phg4 self-assembled into stable β-sheet nanofibers, which was facilitated by π-staking of aromatic side chains alongside hydrogen bonding between backbone amides along the nanofiber axis. As shown in Fig. 2a, in an aqueous medium, β-sheet nanofibers of Phg4 were highly entangled to form a nanofiber hydrogel network. In addition, Phg4 demonstrated a unique surface activity in the presence of immiscible oils and was superior to commercial emulsifiers in stabilizing oil-in-water (O/W) emulsions due to interfacial adsorption of amphiphilic nanofibers.

Peptide-based nanostructures have attracted wide attention in the field of cancer targeting and drug releasing. In fact, peptides have the advantages of simple structure, good biocompatibility, and high chemical diversity [47]. The nanotube structure formed by the self-assembly of peptides has been widely used as a carrier for loading drugs and targeting cancer cells in biomedicine [48–50]. The earliest peptide nanotubes were made up by the self-assembly of D, L-alternating-α-cyclic peptides. For example, D, L-α-peptides and free dipeptides have been assembled into nanotubes by hydrogen bonding between the backbones of peptide molecules [44, 51]. Dipeptides were the smallest structure to synthesize uniform peptide nanotubes via self-assembly. For instance, diphenylalanine motif was a dipeptide, which was a part of the β-amyloid peptide in Alzheimer's disease and could be prepared simply. The peptides formed multi-walled nanotubes with a typical diameter of 80–300 nm and the length of a few µm after dissolving in 1,1,1,3,3,3-hexafluoro-2-propanol (HFIP) solution. When the dipeptide solution was placed onto a substrate, a thin layer of vertically aligned peptide nanotubes can be formed [51]. The rapid evaporation of HFIP may cause supersaturation, thereby promoting the formation of a large number of nucleation sites on the surface. The dipeptide assembled into nanotubes in a unidirectional manner in these nucleation sites. It grew upward toward the liquid–gas interface, and these nanotubes were stable under extreme conditions (such as 121℃, 1.2 atm). Circular 2D chromatography was not changed with the temperature increasing from room temperature to 90℃. However, nanotubes were stable when heated to 150℃, and degraded at 200℃.

Peptides can also be formed as nanotubes by twisting of the laminate bilayers, for example, Ac-KLVFFAE-NH_2_ (Aβ 16–22). Dipak et al*.* used simple synthetic precursors, such as tryptophan and tyrosine, to synthesize poly(amino acid) nanotubes via self-assembly in ethanol [52]. Molecular interactions play important role in the self-assembly formation of aggregate nanotubular structures. The polymer assembly process could be visualized as shown in Fig. 2b. Once the stacking took place to get a flat structure then the drive for minimum surface area spontaneously made it to a cylindrical shape. In addition, the self-assembly of amino acid molecules was mediated by synergistic force of water molecules in solvents (such as ethanol). With the help of hydrogen bonds, water molecules interact with the formed nanotube walls and other water molecules to achieve structural stability. In Fig. 2c, it can be seen that the nanotubes formed from tyrosine exhibited uniform length and diameter. Studies have found that the interaction between amino acid side chains and charged ends also plays a key role in initiating the self-assembly process. In summary, stacking interaction followed by self-assembly readily drive to attain minimum energy on the surface leading to the formation of nanotubes.

#### 2D peptide nanosheets and nanobelts

As typical 2D nanoscale components, nanosheets and nanoribbons provide important physical and chemical connections between low-dimensional components (e.g. nanoparticles, nanofibers, and nanotubes) and extended 3D structures (e.g. crystalline solids). The controlled manufacturing of nanoscale 2D components poses a major challenge to current synthesis methods. Nevertheless, the development of effective strategies for creating 2D components is critical to the success of the emerging field of 2D nanostructures. In particular, chemical and physical principles are applied to design nanosheet structures for integration into functional devices. The molecular programming and self-assembly of sequence-defined peptides provides important prospects for the fabrication of 2D nanostructures. Peptides have high-density chemical functions and can be arranged in defined macromolecular structures. This molecular-level information can be used to guide highly specific intramolecular and intermolecular interactions in a given structural environment, and promote the self-assembly of thermodynamically stable and structure-defined 2D components.

Previous studies have demonstrated that the introduction of structural modifications into collagen-mimic peptides can reproduce the complex pattern of natural collagen assembly. Metal ion coordination, π-π interaction, and disulfide bond formation have been used to guide the high-order assembly of collagen-mimic peptides into supramolecular structures, including fibers, discs, network, and spherical nano-assemblies. However, since it is difficult to reliably predict the assembly mode in consideration of the initial peptide sequence, the resulting assembly usually did not exhibit high degree of order implicit in the structural hierarchy of natural collagen isotypes. These obstacles have been made breakthrough progress in recent studies.

**Fig. 3:**
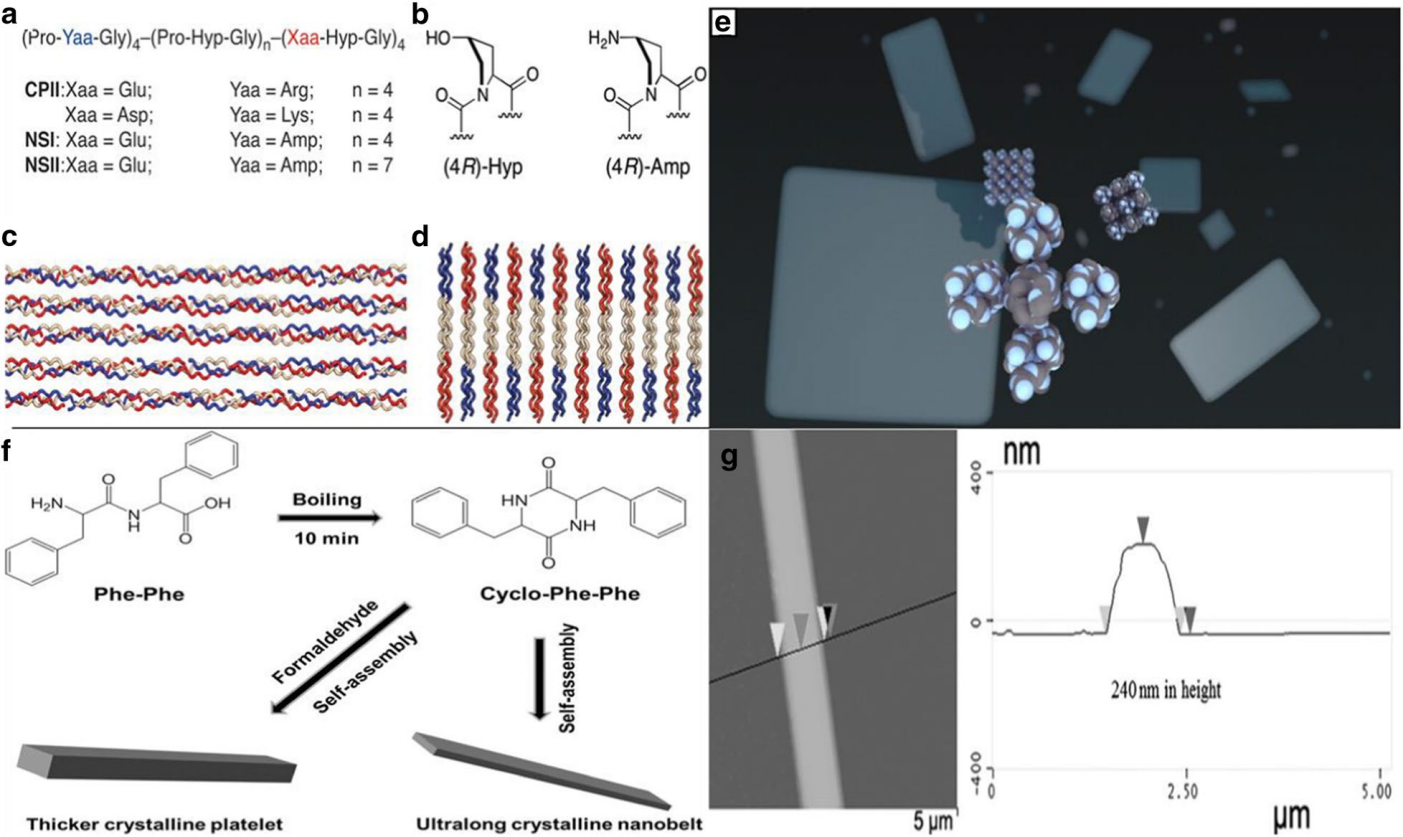
Design and synthesis of 2D peptide nano-assemblies: **a** Amino acid sequences of peptides. **b** Structures and preferred ring pucker conformers of imino acid derivatives. **c** Staggered orientation of peptides within synthetic collagen-mimetic fibrils. **d** Proposed packing of collagen triple helices within 2D layers through electrostatic interactions. **e** The self-assemble into structurally defined nanoscale sheets. Reprinted with permission from Ref. [53], Copyright 2014, American Chemical Society. **f** Schematic formation mechanism of ultralong peptide nanobelts. The thickness of crystalline nanobelts can be increased by the introduction of formaldehyde. **g** AFM image of peptide nanobelt. Reprinted with permission from Ref. [55], Copyright 2016, Wiley–VCH

Jiang et al. reported the design of two collagen-mimic peptide sequences, i.e., NSI and NSII (the sequences are shown in Fig. 3a), which were self-assembled into a structure-defined nanosheets [53]. The underlying structure of the designed nanosheets can be understood by the layered stacking of 2D collagen triple helices. The sequences of peptides NSI and NSII were based on the original design of CPII in that the pattern of positively and negatively charged amino acid residues was maintained to ensure charge complementarity (Fig. 3a). However, in order to direct the Coulombic interactions between triple helices, the positively charged arginine residues in the first four triads of CPII were replaced with the noncanonical amino acid, (2S,4R)-4-aminoproline (Amp) (Fig. 3b). NSI and NSII nanosheets showed very high internal order on a large 2D length scale without introducing unnatural structural interactions. In addition, the thickness of the nanosheets can be controlled by tailoring the molecular length and terminal function. The presence of functionalized end-capping groups provided an opportunity to control surface chemistry to promote specific interactions with complementary functionalized exogenous substrates. The powerful experimental strategy of charge complementation was used to control the supramolecular structure in peptide-based materials and to define the backbone structure of peptide molecules (Fig. 3c, d). Experimental results showed that the collagen triple helix represents a flexible platform that can create extended self-assembled 2D nano-assemblies through structural encoding of electrostatic interactions (Fig. 3e).

The self-assembly path of peptide can also provide the way for the creation of nanobelts and other nanostructures [54]. For instance, long nanostrips with 10–20 nm hight and 150 nm width have been created after two days of cultivation. The formation of various peptide nanostructures depends on the concentration of peptides in the solution. Narrow nanoribbons and twisted nanoribbons were formed at low concentrations and nanoribbons were formed with increasing the concentration. The structure of nanobelts was also affected by the pH value. As the pH value increased, flat nanoribbons can be transformed into grooved nanoribbons. And this process was reversible at low pH.

Besides, the introduction of a specific functional group like the alkyl tail may have a regulating effect on the formation of peptide nanostructures. By changing experimental conditions, the level of hydrophobic interactions and repulsive forces between the peptides can be adjusted to determine the shape of the resulting nanoribbons [44]. Recently, Li et al*.* developed an approach to complete the intramolecular cyclization of linear diphenylalanine (L-Phe-L-Phe, FF) and crystallization in 10 min by solvothermal treatment [55], which can facilitate the phase separation of gels and speed up the kinetics of cyclization reaction and concomitant nucleation, growth and crystallization (Fig. 3f), resulting in the formation of ultralong crystalline peptide nanobelts. By introducing a small amount of aldehydes (such as formaldehyde) into the organogel, the thickness of the nanoribbons can be increased to hundreds of nm (Fig. 3g), endowing them the function of optical waveguides. In addition to the conventional straight peptide optical waveguide, a curved optical waveguide can be realized in a curved peptide crystal when the length of the peptide crystal nanoribbons reaches hundreds of micrometer. The reported approach opens a way for the development of optimal devices toward potential biological applications.

#### 3D peptide vesicles

Vesicles are closed 3D structures, which are mainly spherical. Due to their flexibility, hollow vesicles are usually single-layer or double-layer membranes composed of natural or synthetic amphiphilic molecules [56]. The membrane can isolate and transfer substances and shows good application prospects in drug/gene delivery and nanoreactors. In recent years, substantial progress has been made especially in stimulus–response applications. However, peptides are usually assembled into 1D objects, while peptide-based self-assembled vesicles have rarely been reported [53].. The planarity of the peptide bond restricts the flexibility of the polypeptide chain, the presence of hydrogen bonds impose directionality during assembly, and the chirality of amino acids gives the handedness of the entire self-assembly process. All these effects reduce the flexibility of the polypeptide chain, making it challenging to aggregate peptides into vesicle structures.

**Fig. 4:**
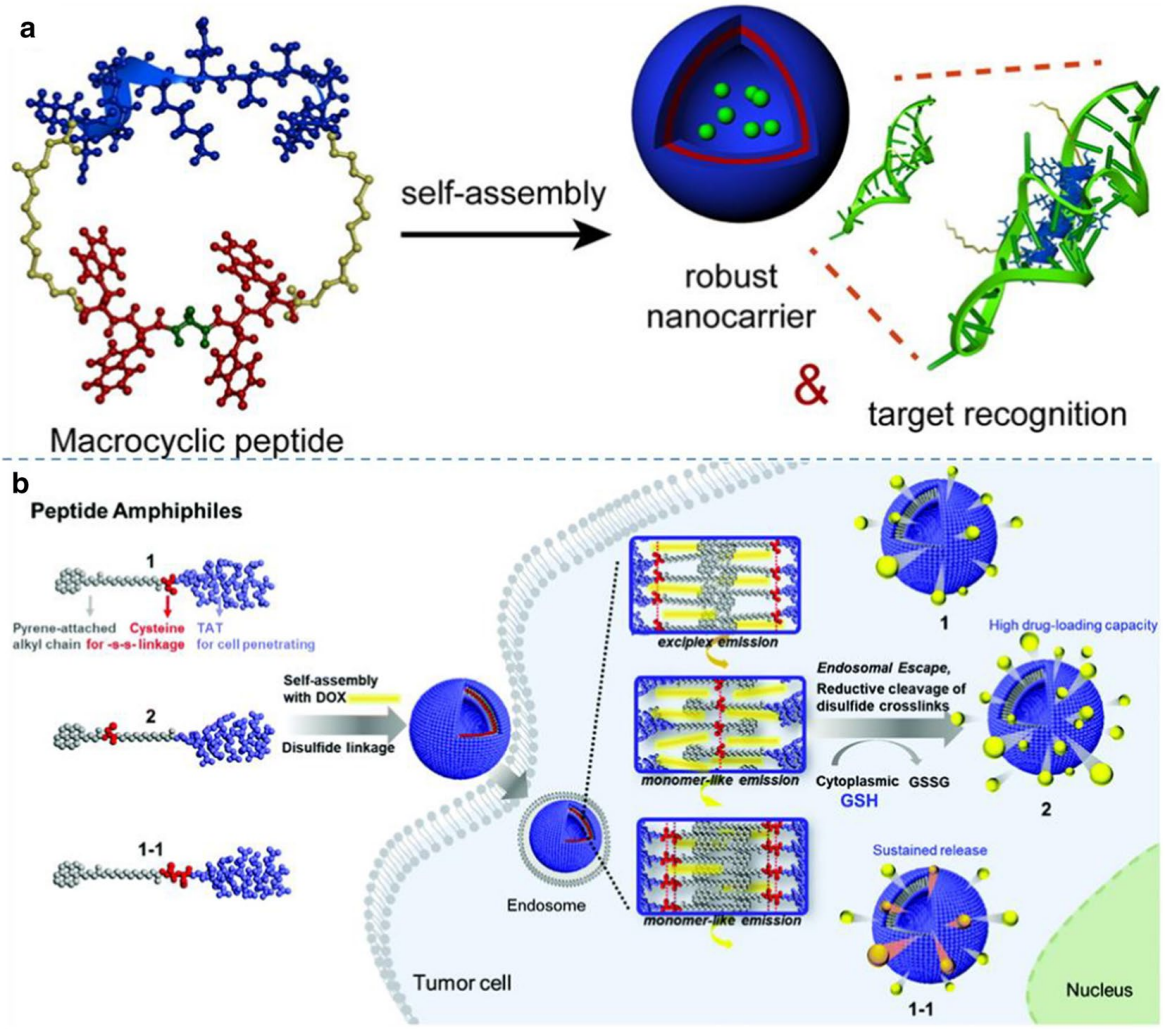
**a** Self-assembly of macrocyclic peptides for robust vesicle formation with RNA molecular recognition capability. Reprinted with permission from Ref. [57], Copyright 2014, American Chemical Society. **b** Schematic representation of the self-assembled peptide vesicles containing mono- or bis-disulfide bridges within the wall. Reprinted with permission from Ref. [58], Copyright 2020, Royal Society of Chemistry.

In a recent study, Jeong and Lim developed macrocyclic peptide building blocks that formed self-assembled peptide vesicles with molecular recognition capabilities [57]. In their study, macrocyclic peptides were significantly different from conventional amphiphiles. They could self-assemble into vesicles at very high hydrophilic-to-total mass ratios. The flexibility of the hydrophobic self-assembly segment is essential for the self-assembly of peptides into vesicle nanostructures. To give the hydrophobic segment flexibility and enhance the π-π interaction, they moved glycine from the N-terminal region to the middle part of the hydrophobic segment. The relocation of a single glycine residue significantly affected the nanostructure morphology and uniformity of the self-assembled peptide nano-assemblies. The obtained results suggested that inserting the pliable amino acid glycine increased the overall flexibility of the hydrophobic chain, which in combination with the reduced mobility of the macrocyclic peptide, reinforced the internal hydrophobic packing of the molecular assembly, as shown in Fig. 4a. The unique features of this peptide vesicle system include homogeneous size distribution, unusually small size, and robust structural and thermal stability. The peptide vesicles exhibited molecular recognition capabilities, in which they selectively bound to target RNA through surface-displayed peptides. This study demonstrated that self-assembled peptide vesicles can be used as strong intracellular delivery vehicles that recognize specific biomacromolecule targets.

Peptide vesicles have been found for promising applications in medicine and materials science engineering. The tremendous opportunity of peptide engineering allows us to use heuristics to design various peptides that form vesicles. Kim et al. reported the glutathione-triggered release of an anticancer drug from vesicles constructed with peptide amphiphiles (PAs) containing a cell-penetrating TAT peptide that was synthesized by varying the position and number of disulfide-linkages in the PAs [58]. As indicated in Fig. 4b, when it came to the self-assembly behavior of PAs, the position of cysteine (C) within the molecular structure affected the resulting morphology. The presence of C residue at the N-terminus of the hydrophilic peptide segment (1) led to the formation of vesicle whereas the location of carbon chain between the pyrenyl group and alkyl chain of the hydrophobic part (2) formed 2D sheets. Upon disulfide bond formation, only vesicles were generated from all PAs. In particular, the disulfide bond had an effect on the loading capacity of hydrophobic drug within the nanostructures by modulating the space for drugs to be loaded. Additionally, the cross-linked vesicles of PA with increased number of C (1–1-ss) led to the more sustained release of anticancer drug upon exposure to GSH confirmed by release profiling and FACS analysis [58].

#### 3D peptide hydrogels

Over the past few decades, hydrogels have received much attention due to their potential applications for tissue engineering, drug release, water treatment. They are a class of materials composed of polymers or low-molecular-weight cross-linked hydrogels, which can fix large amounts of water or aqueous solutions while maintaining a unique 3D structure [59]. The high-water content and adjustable properties of hydrogels make them suitable as synthetic mimics for soft tissue microenvironments, as well as promising media for local storage and delivery of therapeutic agents.

Besides, controlled supramolecular non-covalent interactions or the chemical covalent bond that acted on hydrogels improved physical, chemical, or biological stimuli properties of hydrogels. Peptides have the advantages of high biocompatibility, versatility, and adjustable secondary structure. The secondary structure, especially the α-helix β-sheet conformation, can be used as a driving force to form a uniform fiber structure and further assemble into the covalently cross-linked 3D network, which can encapsulate water media to create a hydrogel. Previously, peptides have been widely used as versitale molecular building blocks for the preparation of hydrogels [59, 60].

**Fig. 5:**
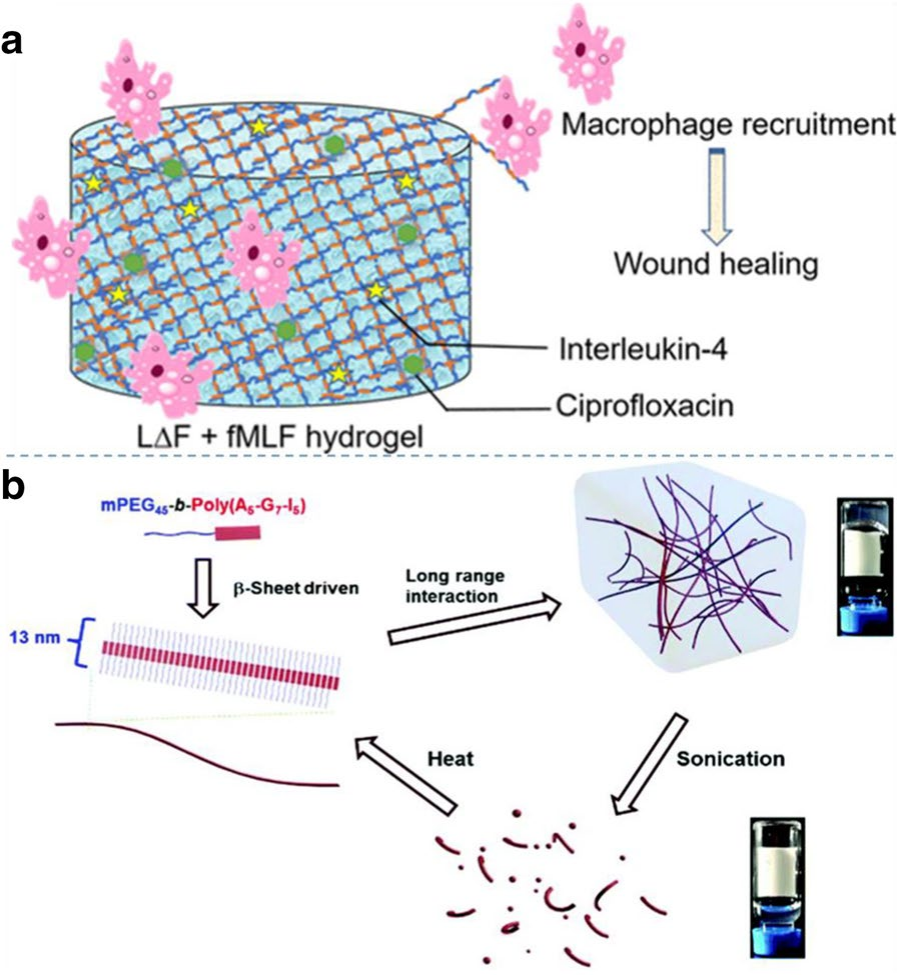
Design and synthesis of peptide hydrogels: **a** A co-assembly strategy to generate an injectable ultrashort bioactive peptide hydrogel formed by mixing a dipeptide hydrogelator. Reprinted with permission from Ref. [61], Copyright 2020, American Chemical Society. **b** Thermal preparation of peptide hydrogels. Reprinted with permission from Ref. [62], Copyright 2017, Royal Society of Chemistry

Thota et al. reported a co-assembly strategy for generating injectable ultrashort bioactive peptide hydrogels [61], which could be used as a novel material for wound dressing. The designed hydrogel is composed of dipeptide hydrogelator LΔF and fMLF (Fig. 5a). Macrophage recruitment plays a crucial role in wound healing, where the M2 phenotype secretes multiple growth factors (GFs), to attract other cells, such as fibroblasts, to help wound healing. One such strategy was to ensure that endogenous GFs were spontaneously and continuously provided to wounds, avoiding proteins recombinant in vivo. This strategy is safe and can be rapidly degraded in the body, but it is limited by the high production cost, and further exploration of better production methods is needed. Recently, Fan et al*.* reported a peptide-based hydrogel system [62]. A polypeptide hydrogel system prepared from ternary sequences of l-alanine, glycine and l-isoleucine within a polypeptide segment of a PEG-peptide diblock structure exhibited thermo-, mechano- and enzyme-responsive properties, which enabled the encapsulation and release of naproxen (Npx) (Fig. 5b). The created hydrogel can encapsulate and release Npx within 6 days, which illustrates the potential application of this peptide hydrogel as an injectable local delivery system for small molecule drugs.

### Peptide-based hybrid assemblies

In addition to the formatio of pure peptide nano-assemblies, the formation of peptide-based hybrid assemblies can be achieve by controlling the self-assembly of various conjugates of peptide with other components, including alternative peptide, polymers, nanoparticles, 1D nanomaterials, and 2D materials such as graphene.

#### Peptide-peptide hybrids

The hierarchical self-assembly capabilities of peptides have been extensively explored to design functional biomaterials. Peptides have been shown to adopt a variety of different nanostructures, such as vesicles, micelles, fibers, bands and tubes. The supramolecular assembly of peptides is mainly affected by non-covalent interactions between molecules, such as hydrophobicity, van der Waals force, electrostatic, hydrogen bonding and π-π interaction. Peptide-peptide binding interactions are known to be determined by the sequential and conformational structures, as well as surrounding environments [63].

Both experimental and theoretical efforts have revealed complex interaction characteristics. However, due to the heterogeneity of the peptide-peptide connection structure, ambiguities and challenges arise in the relevant research. To face these challenges, Zou et al*.* proved that a significant cooperative binding effect can be identified as the amino acid site determining the binding properties of peptide interactions [63]. A flow cytometry (FCM) measurement method based on microbeads was introduced to determine the binding affinity of peptides under constant experimental conditions. The tryptophan scanning mutation analysis of 14 peptides containing glycine provides a mapping of binding energy sites. Specifically, when the two tryptophans are located in different positions, the characteristic of cooperative binding can be observed, which depends on the relative positions of the two tryptophans. The best separation of 6–10 amino acids between the two binding sites can be determined to achieve maximum binding. The observed variations in the cooperative inter peptide binding characteristics, as reflected in pronounced position dependent interaction strength in this study, may provide a basis for a more thorough analysis of the conformational effects of peptide structures.

Peptide-peptide hybrid therapy has been one of the most important treatment strategies in clinical practice and has been widely used in many diseases, including cancer and acquired immunodeficiency syndrome (AIDS). Recently, a co-administration strategy of an anticancer peptide and a KLA peptide modified by a penetrating peptide has been studied to explore the anticancer effect and the mechanism of peptide-peptide synergy. Hu et al. designed a peptide-peptide co-administration therapy between the hybrid peptide KLA-TAT and the cationic anti-cancer peptide HPRP-A1 to increase the anti-cancer activity of the combined peptide through a synergistic effect [64]. KLA is a pro-apoptotic peptide that can destroy the mitochondrial membrane during cell internalization, thereby inducing rapid apoptosis of cancer cells. TAT can help other molecules enter the cell through the endocytic pathway.To enhance the anti-tumor ability of hybrid peptides, TAT-modified KLA was designed and synthesized as a highly effective anti-cancer peptide. Another cationic membrane active peptide, HPRP-A1, can destroy the integrity of the cancer cell membrane, resulting in the loss of cell membrane barrier function and other molecules can easily enter the cell. Therefore, a strong synergistic effect was expected via the combination of KLA-TAT and HPRP-A1. After the two peptides worked together, the cell membrane can be more severely damaged, and more lactate dehydrogenase (LDH) can be released from the cytoplasm. The study of the sequence and composition effects of the peptide structure in the peptide-peptide connection provides a new way for disease treatment and prevention.

**Fig. 6:**
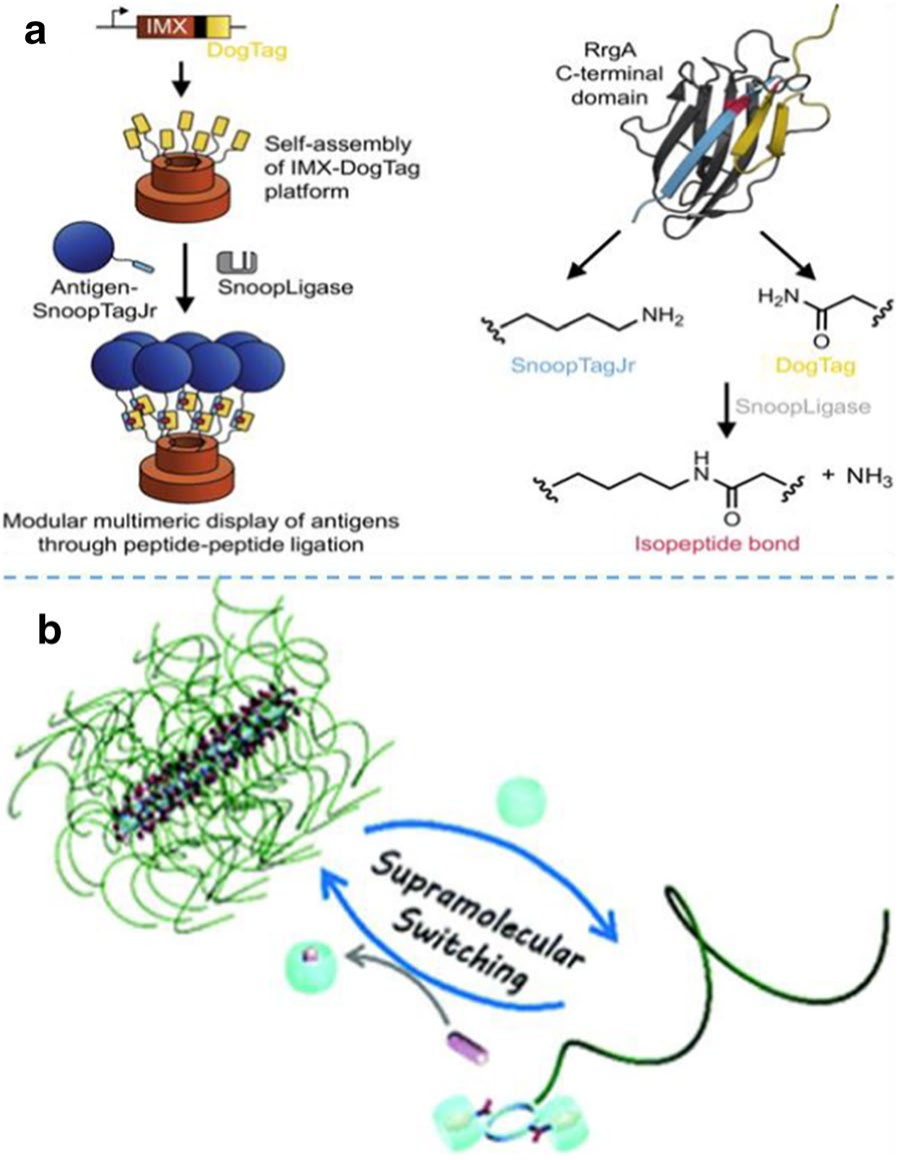
Design peptide-molecule hybrids for the synthesis of peptide hybrid nano-assemblies: **a** Peptide-peptide system for self-assembly. Reprinted with permission from Ref. [65], Copyright 2019, Nature Publishing Group. **b** Peptide–polymer conjugate via host–guest chemistry for self-assembly. Reprinted with permission from Ref. [67], Copyright 2019, American Chemical Society

At present, for many infectious diseases there is still no vaccine, even though potential protective antigens have been identified. There is an urgent need for suitable platforms and coupling pathways to transform these antigens into vaccines with broad protection and scalability. Andersson et al*.* applied a newly established peptide-peptide ligation approach, SnoopLigase, for specific and irreversible coupling of antigens onto an oligomerization platform [65]. SnoopLigase was engineered from a Streptococcus pneumoniae adhesin and enables isopeptide bond formation between two peptide tags: DogTag and SnoopTagJr. The introduction of SnoopLigase to the hybrid peptide system would lead to covalent coupling of DogTag to SnoopTagJr through isopeptide bond formation, enabling modular oligomerization of the antigen (Fig. 6a). The use of peptide-peptide conjugation for antigen oligomerization platform is scalable for vaccine platforms and reveals high potential in biomedical application.

#### Peptide-polymers hybrids

Although the peptide-polymer combination therapy is clinically considered as a potential cancer treatment option, the current treatment results are far from perfect. Different anti-cancer drugs and inhibitors have different pharmacokinetics, biodistribution, and membrane transport properties, resulting in inconsistent circulation and cellular uptake of different drugs. Using a non-optimal drug combination at the tumor site can compromise the synergistic effect provided by the combination therapy. Therefore, encapsulating multiple drugs in a nanocarrier can promote the simultaneous delivery of drugs at the tumor site in a precise combination ratio.

Ovarian cancer shows the highest mortality rate among all gynecological cancers in the world. Paclitaxel (PTX) is a mitotic disruptor and is currently the standard clinical treatment for ovarian cancer. However, the frequent occurrence of multidrug resistance (MDR) is a common obstacle to effective chemotherapy, leading to the failure of chemotherapy in most ovarian cancer patients. One of the promising strategies to overcome tumor multidrug resistance (MDR) is to provide both anticancer drugs and P-glycoprotein (P-gp) inhibitors. To enhance the cancer cellular internalization and implement the controlled drug release, an iRGD peptide-modified lipid-polymer hybrid nanosystem (LPN) was fabricated by Zhang and co-workers [66], which exhibited the loading of paclitaxel (PTX) and tetrandrine (TET) at a precise combination ratio.

In addition to the acceleration of the apoptosis for cancer cells, peptide-polymer conjugates have aroused great interest in the production of functional materials due to their selectivity and synergy [67, 68]. For example, supramolecular polymers have recently attracted widespread attention, which are defined as the polymer arrays of monomer units held together by highly oriented and reversible non-covalent interactions, such as multiple hydrogen bonds, host–guest interactions, metal coordination interactions, and interaction with donor–acceptor. Among them, tubular supramolecular polymers assembled from cyclic peptide-polymer conjugates belong to a relatively new class of self-assembled supramolecular polymers.

Song et al. designed and synthesized a cyclic peptide conjugated with a water-soluble polymer-poly (ethylene glycol) (PEG), which could form tubular supramolecular polymers in aqueous solution (Fig. 6b) [67]. It has been reported that Cucurbiturils (CB) binds strongly with a phenylalanine moiety, with the binding constant as high as 106 M^−1^. Two phenylalanine was introduced on the cyclic peptide, which can non-covalently bind two bulky CB host molecules to each cyclic peptide polymer conjugate. Moreover, a competitive guest molecule (1-adamantanamine, ADA) was added to disassociate the binding between the cyclic peptide–polymer conjugate and CB. In this way, the self-assembly of the cyclic peptide-polymer conjugate can be reversibly regulated. Considering that various functional polymers can be combined with cyclic peptides to construct various functions of tubular supramolecular polymers, the proposed strategy provided a new perspective on the preparation of finely controlled tubular structures and functionalized supramolecular polymers [68]. This peptide-based hybrid material has a sensitive response to various biological stimuli and was easy to integrate multiple biological functions such as cancer cell diagnosis and targeted drug delivery, exhibiting broad application prospects in biomedical community.

#### Peptide-0D materials hybrids

Hybrid materials of 0D materials (quantum dots and nanoparticles) and peptides have been widely explored as general building blocks for various applications, such as biomedical scaffolds, tissue regeneration, biomedicine, and nanotechnology. There are 20 kinds of natural amino acids that can be used for peptide synthesis, which provides many possibilities for the design and synthesis of functional materials. As we all know, the motif design of peptide molecules plays an important role in the synthesis of functional peptide hybrid materials.

Recently, the design of a novel functional peptide molecule was reported [69], which has the abilities to form peptide nanofibers (PNFs) and recognize with graphene quantum dots (GQDs) and graphene oxide (GO) nanosheets specifically. Based on the design of peptide sequence, the ternary GQD‐PNF‐GO nanohybrids were successfully synthesized. Subsequently, to understand the formation mechanism of this ternary nanostructure, they further studied the interaction between GQD, PNF and GO through atomic foce microscopy (AFM)-based force spectroscopy and fabricated biosensors. The manufactured biosensor exhibited high sensitivity and selectivity, low detection limit and wide linear range for H_2_O_2_ detection. The proposed strategies shown in this work, such as peptide motif design and self-assembly on GQDs, benefits to further creation of functional peptide materials and understanding of their self-assembly mechanism.

Biological enzymes have recently been proved to be an effective means for directing supramolecular self-assembly in a temporal and spatial manner. In the manufacture of certain biological materials, hybrid self-assembly of 0D materials such as peptides and nanoparticles plays an important role [70]. Miryam et al. reported the design of a hybrid supramolecular hydrogel prepared from the Fmoc–FFpY tripeptide (F: phenylalanine, Y: tyrosine, p: phosphate group) and silica nanoparticles (NPs) that functionalized by alkaline phosphatase (AP) covalently [71]. As an efficient hydrogelator, AP transformed Fmoc–FFpY into Fmoc–FFY, which was able to self-assemble spontaneously and exclusively from the surface of nanoparticles, giving rise to the formation of a homogeneous nanofibrous network. They further proved that this dense network was due to not only the attractive interaction between nanoparticles and AP, but also the strong interaction between self-assembled nanofibers and enzymes covalently immobilized on nanoparticles.

**Fig. 7:**
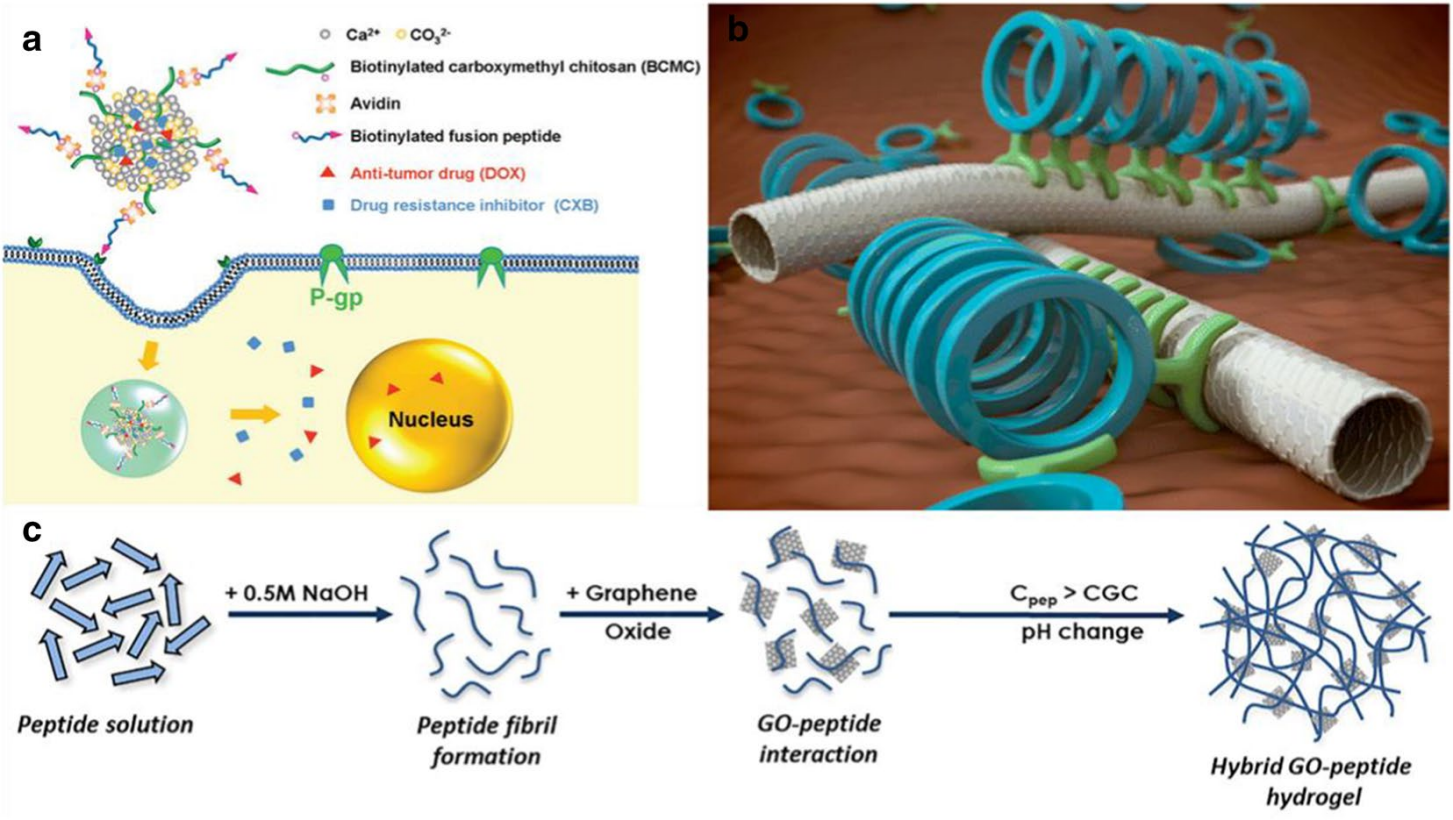
Design of peptide-material hybrids for the synthesis of hybrid nano-assemblies: **a** Peptide-nanoparticle hybrids for synergistic drug delivery. Reprinted with permission from Ref. [72], Copyright 2017, Royal Society of Chemistry. **b** Peptide-SWCNTs hybrids for self-assembly. Reprinted with permission from Ref. [74], Copyright 2014, American Chemical Society. **c** Peptide-GO hybrid hydrogels. Reprinted with permission from Ref. [77], Copyright 2019, Elsevier Ltd

Peptide-nanoparticle combination therapy has received increasing attention on medicine, which not only exerts the synergistic effect of multiple treatment methods, but also overcomes the multi-drug resistance in cancer treatment. Wu et al*.* developed a simple self-assembly strategy to modify polymer/inorganic hybrid nano-sized drug delivery systems with functional peptides [72]. To enhance drug delivery efficacy and overcome tumor drug resistance, a functional fusion peptide containing an RGD sequence for tumor targeting and an R8 sequence for cell penetration was introduced onto the surface of biotinylated carboxymethyl chitosan/CaCO_3_ (BCMC/CaCO_3_) hybrid nanoparticles through biotin–avidin interaction to obtain peptide functionalized nanoparticles (PNP) (Fig. 7a). The peptide functionalization resulted in improved delivery efficiency and effective inhibition for drug resistant tumor cells. Co-delivery of an anti-cancerous drug (doxorubicin hydrochloride, DOX) and a cyclooxygenase-2 inhibitor (celecoxib, CXB) by PNP further improved the therapeutic efficiency by effectively down-regulating P-gp expression to reduce P-gp mediated drug efflux and increase intracellular drug accumulation.

#### Peptide-1D material hybrids

Combining 1D materials such as carbon nanotubes (CNTs), nanowires and biological suprastructures to form hybrid functional components is an innovative research field with broad application prospects in the fields of medicine, nanotechnology, and materials science. Combining the mechanical and electronic properties of 1D materials, the properties of protein molecular recognition and catalytic activity provide opportunities for chemists, biologists and materials scientists to understand and develop new nanomachines, sensors, or other molecular combinations. The role of peptides is to dissolve CNTs and can be classified according to diameter or chirality. In turn, CNTs can support and immobilize enzymes to create functional materials. Other applications include using peptide to assemble ordered hierarchical objects containing CNTs, and using CNTs that serve as peptide vaccine carriers. Based on this, peptide-CNT hybridization can form biological scaffolds, which have been used as therapeutic and imaging materials [73].

Currently, hybrid materials composed of single-walled carbon nanotubes (SWCNTs) and self-assembled cyclic peptide nanotubes (SCPNs) have been reported. For example, Montenegro et al*.* reported the preparation of a dual composite material in an orderly combination of two tubular elements [74]. The coupling model between SCPN and SWCNT is shown in Fig. 7b. In this structure, SWCNTs were used as the template for SCPN, enabling SWCNTs and SCPNs to form noncovalent bonds through pyrene paddle. The resulting special hybrids formed highly ordered directional arrays that exhibit complementarity, such as electrical conductivity. The attachment to the π surface of CNTs stabilized the assembly of peptide nanotubes, and the hydrophilic side chains on the attached SCPNs in turn stabilize the CNTs in aqueous solution. This parallel dual-tube disposition should allow for the alignment (along the CNT) of molecules of interest that would be attached to the cyclic peptide ring or even facilitate possible double flow transport of different ions or molecules through the cavity of each nanotube. The SWCNT/SCPN hybrids obtained in this work also formed highly ordered oriented arrays when deposited on glass or mica.

In addition to CNTs, nanowires are of great interest due to their potential biological applications, such as drug delivery or sensing devices. Li et al*.* reported the development of a unique cell capture and release platform based on GaN semiconductor nanowires [75]. First, the GaN nanowires were synthesized using chemical vapor deposition. After that, peptides were introduced on the surface of the nanowires. Subsequently, the biotinylated aptamer was linked to the peptide via streptavidin. After the peptide hybrid module is treated with sodium chloride solution, the peptide may be desorbed from the surface. This indicated that the reversibility of the binding of the peptide to the surface allows the substrate to be reused. Finally, the functionalized surface can be regenerated by repeating the above process. Overall, the inding processes are reversible, which is not only useful for downstream analysis but also for reusability of the substrate.

#### Peptide-2D material hybrids

Tumor resection is widely employed to prevent tumor growth. However, the defective tissue at the original tumor site can also cause tissue or organ dysfunction, thereby reducing the patient's quality of life. Therefore, it is very important to regenerate tissues and prevent tumor recurrence. Based on the concept of "kill first and then regenerate", a scaffold-based multifunctional tissue engineering strategy has been developed [76], in which β-tricalcium phosphate (β-water/poly(lactic-glycolic acid copolymer)/dichloromethane emulsion-TCP), 2D black phosphorus (BP) nanosheets, low-dose DOX, and high-dose osteogenic peptides were combined together to form a porous nanocomposite scaffold with hierarchical structure and high mechanical strength. The fabricated scaffold has multiple functions, which can treat bone tumors, resection, induce tissue defects and other diseases, enabling the elimination of tumor cell recurrence inhibition and improve tissue regeneration.

Cell-based cancer treatment methods show great promise in tissue engineering, while one of the key challenges is cell delivery and retention. In the past years, great efforts have been made to design injectable biomaterials for containing and transporting cells at the injury site. Recently, the hybrid assembly of biomaterials and inorganic materials, such as peptide-graphene hybrid, have provided insights for cell drug delivery and tumor treatment. These hybrid nanomaterials composed of self-assembled peptides with customized functions were widely used in nanotechnology, tissue engineering, and biomedical engineering due to their unique structure and characteristics. For example, short self-assembling peptide hydrogels (SAPHs) have attracted great interest since they can mimic the natural extracellular matrix and have great prospects for the design of cell microenvironments from the beginning. Ligorio et al*.* added GO into the peptide self-assembly system during the preparation of peptide hydrogels [77], as shown in Fig. 7c. The binding process of this peptide and GO has two important characteristics. The first one is that at lower pH values, the peptide has an overall positive net charge, and the surface of the graphene oxide flakes have a negative net charge. Therefore, the electrostatic interactions occur between peptides and GO nanosheets to promote good interfacial adhesion between nanofibers and GO. The other characteristic is that this peptide does not have a total net charge at neutral pH, and can be formulated into a stable hydrogel with low mitotic activity suitable for cell culture. The strong interaction between peptides and GO flakes affects the overall properties of the final hybrid hydrogel. The incorporation of GO can improve cell viability and metabolic activity, as well as the mechanical properties of the hydrogel, making it a good candidate for drug delivery in cancer treatment. Due to its unique structure and characteristics, this composite hydrogel has great potential as a stent for injecting nasopharyngeal carcinoma. It is expected to be widely used in the field of tissue engineering and biomedical composites in the future [77].

In another study, a peptide-mediated biomimetic strategy was adopted by Su and co-workers to create the multifunctional 3D graphene foam (GF)-based hybrid minerals [78]. Firstly, 2D peptide nanosheets (PNS) was obtained via self-assembled motif-specific peptide molecules (LLVFGAKMLPHHGA) which are expected to exhibit biological functions, such as biomimetic synthesis of hydroxyapatite (HA) minerals. Subsequently, noncovalent combination of PNS and GF support was applied to form a 3D GF-PNSs hybrid scaffold, which was suitable for the growth of HA minerals. The resultant GF-PNSs-HA minerals exhibited high surface area and interconnected macroscopic and mesoporous structures, which can realize vascularization and flow and transportation of nutrients. The high mechanical strength, ultra-light weight, and shape plasticity of this hybrid 3D minerals make it an excellent platform for building custom, flexible, and stretchable platforms for drug delivery and bone tissue engineering.

## Peptide nano-assemblies for cancer diagnosis

In the above sections, we demonstrate and discuss the self-assembly formation of various peptide-based pure and hybrid nano-assemblies that related to biomedicine and cancer therapy. In this part, the synthesized peptide nano-assemblies for cancer diagnosis, such as fluorescent, magnetic, and photoacoustic imaging as well as specific biosensing of cancer cells, are summarized.

### Fluorescent imaging of cancer cells

Fluorescence microscopy is an essential experimental technique with the properties of high sensitivity, strong specificity, and minimally invasiveness [79]. It can be used in the field of daily life and scientific research. Therefore, it is widely used in biomedical research, focusing on cancer prevention, early detection, and timely treatment. For the fluorescent imaging of cancer cells with peptide nano-assembles, rogust fluorescent probes should be introduced onto the peptide materials.

The first type of fluorescent labels for cancer imaging is the fluorescent groups that bound onto peptide nano-assemblies. For instance, Fan et al. demonstrated that the formation of tryptophan-phenylalanine (Trp-Phe) nanoparticles transferred the intrinsic fluorescence signal of the peptide from ultraviolet to the visible range [80]. Visible emission signals allowed dipeptide nanoparticles (DNPs) to be used as imaging and sensing probes. Dipeptides composed of natural aromatic amino acids, including Trp, Tyr (tyrosine), or Phe, which were used as the basic units to synthesize fluorescent DNP through π-π stacking, and coordination of metal-optimized fluorescent properties of peptide assembly range from UV to visible. The obtained results indicated that DNPs have the characteristics of biocompatibility, visible fluorescence, and photostability. Besides, MUC1 aptamers could be utilzied to modify DNPs to form conjugates, which can be used as fluorescent probes for cancer targeting and sensing, real-time imaging and drug release monitoring. Hu and co-workers reported a CD133-targeting peptide conjugate with NIR-II emitting fluorophore, which was utilized a molecular probe for cancer imaging [81]. In another study, they developed the pH-triggered self-assembly of a designed peptide conjugation with hydrophilic peptide (sequence: SKDEEWHKNNFPLSP) and hydrophobic signal molecule (carboxylated tetraphenylethylene), which exhibited improved signals after peptide self-assembly[82] After the self-assembly formation of peptide nanofibers via pH adjusting, the fluorescent signals could be enhanced significacently. Therefore, the designed peptide nano-assemblies could be used for in vivo imaging, drug delivery, and cancer therapy.

**Fig. 8:**
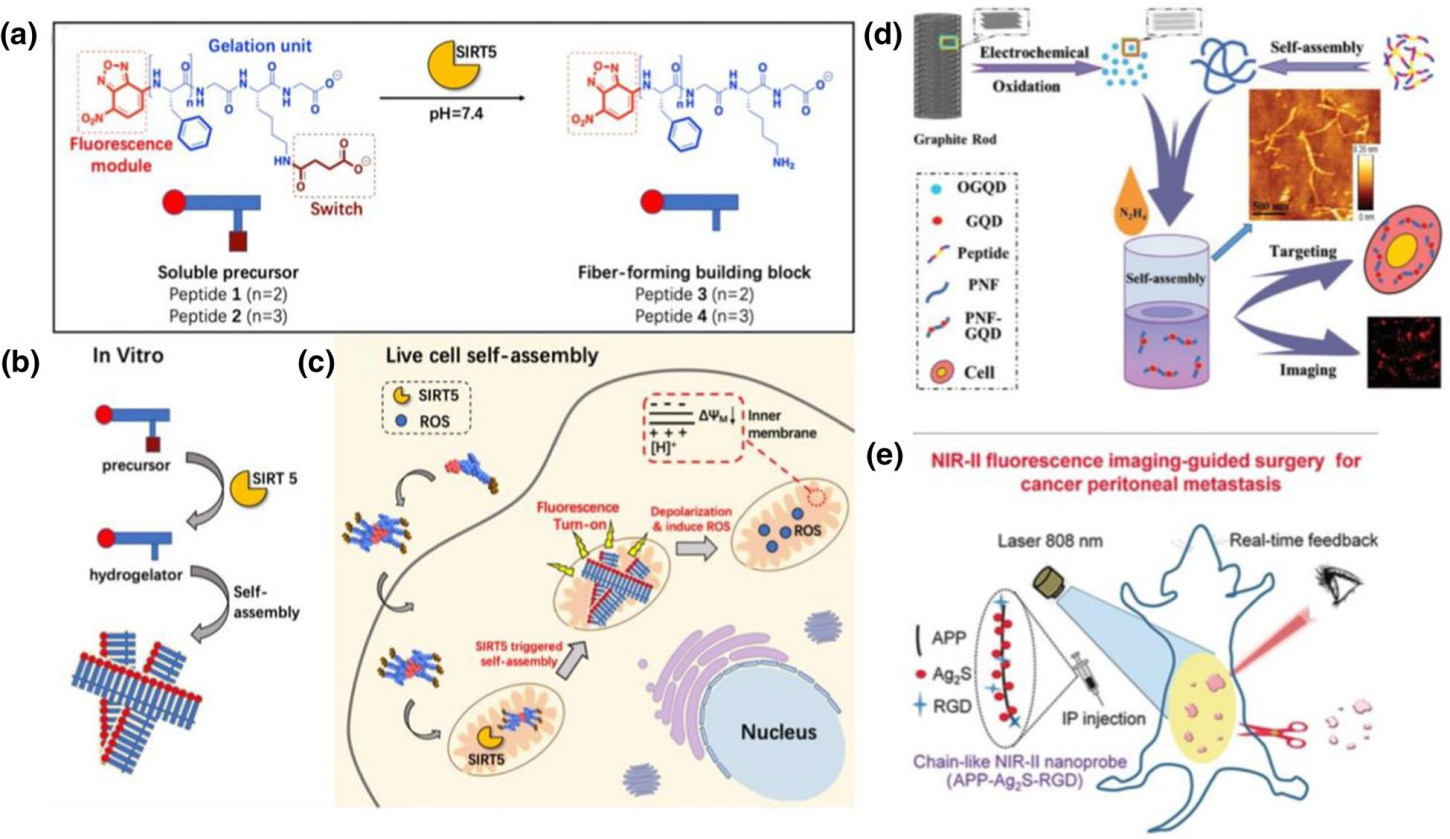
Peptide nano-assemblies for fluorescent imaging of cancer cells: **a**–**c** Design of fiber-forming peptide for live cell imaging. **a** peptide design, **b** self-assembly in vitro, and **c** self-assembly in live cell for imaging. Reprinted with permission from Ref. [83], Copyright 2020, American Chemical Society. (d) PNF-GQD nano-assemblies for imaging. Reprinted with permission from Ref. [84], Copyright 2015, Wiley–VCH. **e** Self-assembled peptide-Ag2S chain for NIR-II fluorescence imaging of cancer cells. Reprinted with permission from Ref. [85], Copyright 2019, Wiley–VCH

Recently, Yang and co-workers reported the desuccinylation-triggered formation of peptide nanofibers for liver cell imaging[83] As shown in Fig. 8a, a fluorescence module is conjugated onto self-assembling peptide sequence for the design of a functional peptide molecules, which could self-assemble into nanofibers at pH 7.4 under the catalysis of SIRT5, mitochondria-localized enzyme. The in vitro self-assembly of the designed peptide molecules was catalyzed by SIRT 5, to form supramolecular nano-assemblies (Fig. 8b). The self-assembly of peptide and formation of nanofibers in mitochondria under the catalysis of SIRT5 enhanced the fluorescent signals in cells, and therefore can be used for highly effective fluorescent imaging of cancer cells (Fig. 8c).

Fluorescent quantum dots and nanoclusters can also be conjugated with peptide nano-assemblies for cancer imaging. As a new type of fluorescent nanomaterial, GQDs showed extraordinary characteristics related to strong quantum structure and edge effects. GQDs had tunable wavelength, stable photoluminescence, and excellent biocompatibility [84]. In addition, GQDs can be used as fluorescence probes in biological imaging. Peptide nanofibers are only a few nanometers thick and 1–1000 nm long. They are biocompatible, rheological, and other features, and are 1D peptide nano-assemblies. Previously, Su et al. developed a new type of peptide-GQ hybrid nano-assemblies that can simultaneously target and display cancer cells [84]. They designed a novel peptide (RGDAEAKAEAKYWYAFAEAKAEAK) with three functional motifs, and prepared PNFs by controlling the self-assembly of the motif-designed peptides. Based on this functional assembly, the synthesized PNFs through non-covalent binding between GQDs and PNFs to form PNFs-GQD nano-assemblies, as shown in Fig. 8d. The obtained results showed that these nano-composites were ideal tools for fluorescent labeling and imaging of tumor cells. In a futher study, they synthesized gold nanoclusters (AuNCs) on the motif-designed peptide nanofibers via biomimetic metallizationn for temperature sensing and cellular imaging [86]. It was found that the formation of peptide nanofibers greatly enhanced the luminescence of AuNCs for nearly 70-fold with 21.3% quantum yield.

In addition, chemical conjugation of quantum dots onto functional peptide chains could be applied for cancer imaging. For instance, Wen and co-workers demonstrated a novel nanoprobes by functional modification of self-assembled amphiphilic peptide (APP) nanofibers with NIR-II Ag_2_S QDs and tumor-targeting RGD peptide sequence [85], as shown in Fig. 8e. The obtained results indicated that the designed peptide nanofiber-based nanochian probe exhibited higher capability for cancer cell detection by comparing with RGD-Ag_2_S QDs. Under NIR-II fuorescent imaging condition, the tumor metastatic foci with a tiny size of 0.2 mm could be observed clearly, which shows high potential of the synthesized nanoprobe for early diagnosis of tumors.

### MR imaging of cancer cells

MR imaging (MRI) is one of the critical diagnostic methods in medical radiology. The molecular weight of reagent increased by combining with the preparation of protein, polymer, and micelle. Thereby relaxivity will enhance as the rotational correlation time increase, and then the correlation coefficient of the MR reagent increases, revealing a significant contrast over a long period.

Bull and co-workers used self-assembling peptide amphiphiles (PAs) to improve the relaxivity of new MR reagents [87]. PAs can self-assemble into cylindrical nanofibers and reveal biological activity and biocompatibility. In their study, Fmoc solid-phase peptide synthesis (SPPS) technology was used to prepare PAs with a branched structure under 0.1 mmol. The structure and architecture of PAs allowed the self-assembly to start in water, which increased the rotational correlation time. They integrated the active functions of other organisms to change the biological activity of amino acid, and achieved high-resolution 3D MRI of cells in vivo. In another case, Chen et al. demonstrated that tumor-targeted multifunctional protein-based nanoparticles could be self-assembled by drug induction [88]. This therapy method does not require complicated chemical or material processing via the combination of multi-model imaging-guided cancer therapy. Using albumin as tumor-targeting nanoparticles to achieve photodynamic/chemotherapy combined targeted tumor therapy HAS (human serum albumin) was pre-modified with light-sensitive molecules and tumor-targeting peptides.

**Fig. 9:**
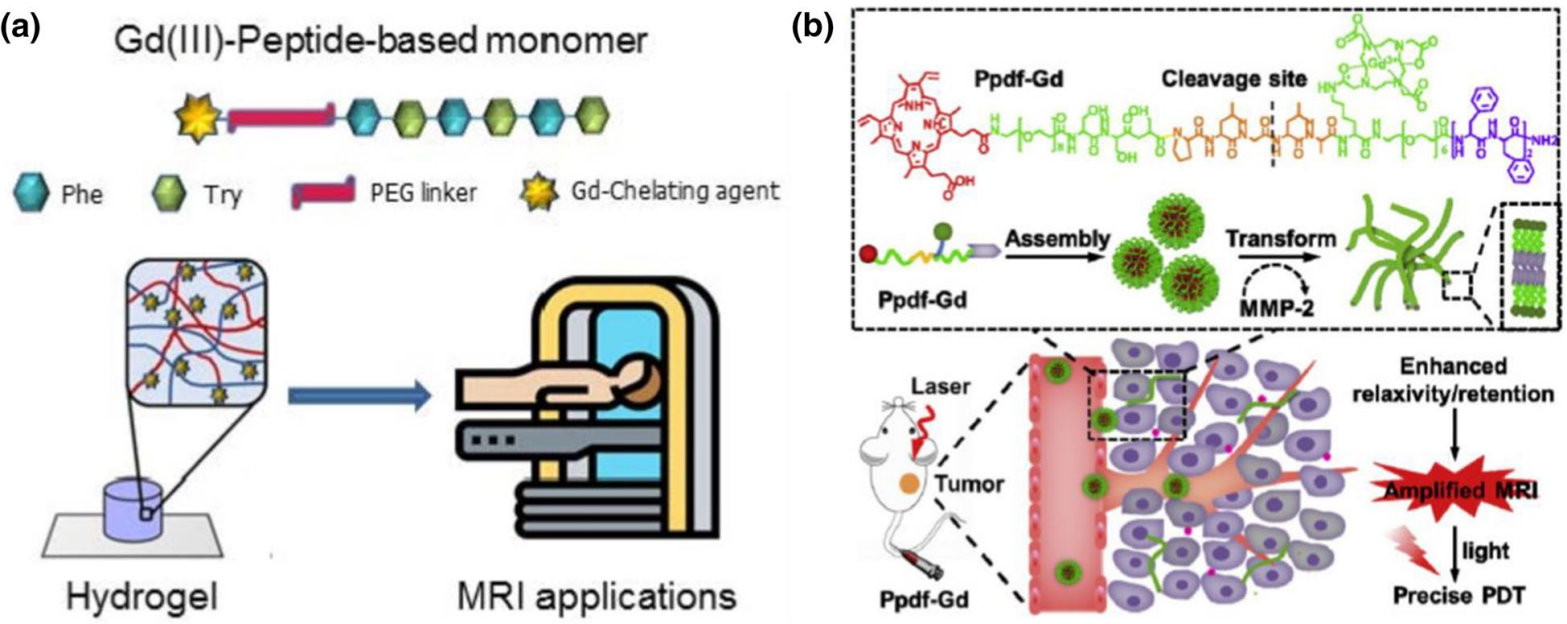
Peptide nano-assemblies for MRI of cancer cells: **a** Gd-modified peptide hydrogel for MRI. Reprinted with permission from Ref. [90], Copyright 2020, MDPI publishing. **b** Gd-modified peptide nanofibers for tumor MRI imaging. Reprinted with permission from Ref. [91], Copyright 2018, Elsevier

To promote the application of peptide-based nano-assemblies for MRI imaging, gadolinium complexes have been usually conjugated to self-assembled peptide nanomaterials for creating MRI contrast agents [89]. For instance, Gallo et al*.* reported the modification of peptide-based soft hydrogels with gadolinium (Gd) complexes for MRI applications [90]. As shown in Fig. 9a, peptide monomer with self-assembling ability is conjugated with Gd(III) via a PEG linker, and the Gd-peptide monomer can self-assemble into nanofibrous hydrogel. In their study, diethylenetriaminepentaacetic acid (DTPA) and 1,4,7,10-tetraazacyclododecane-N,N,N,N-tetraacetic acid (DOTA) were utilized to coordinate with Gd to form DTPA(Gd) and DOTA(Gd), which were further used to design two Gd-peptide monomers, DTPA(Gd)-PEG8-(FY)3 and DOTA(Gd)-PEG8-(FY)3, respectively. The formed peptide-based Gd nano-assemblies could serve as excellent supramolecular diagnostic agents for MRI. In another case, Zhang and co-workers reported the design of a chimeric peptide for dual-stage-amplified MRI through tumor-mediated conformation transition [91], as shown in Fig. 9b. The peptide, Ppdf-Gd, with a sequence of PpiX-PEG8-SSSPLGLAK (DOTA)-PEG6- F4 was designed, which showed self-assembly ability to form spherical nanoparticles in physiological conditions. However, under the catalysis of MMP-2, the created peptide nanoparticles could transform into nanofibers, which enhanced the relaxivity and retention of created contrast agent in tumor region and promoted the MRI of tumors.

In addition to Gd, anticancer agent has been conjugated with peptide nano-assemblies for MRI of cancer cells. For instance, previously Yuan and co-workers reported the design of a novel peptide molecule by conjugating anticancer agent olsalazine (Olsa) with the cell-penetrating peptide sequence RVRR, which could self-assemble into large intracellular nanoparticles under the catalysis of tumor-relevant enzyme, furin [92]. Due to the exchangeable Olsa hydroxyl protons, the created Olsa-RVRR nanoparticles could be readily imaged with chemical exchange saturation transfer MRI technique. The designed peptide-furin hybrid nanoparticles exhibited great potential for imaging cancer cells, drug accumulation, and therapeutic response.

### Biosensing of cancer cell

With the development of biotechnology and nanotechnology, functional biosensors have shown huge potential in practical application. Biosensors based on enzymes and aptamers have been extensively studied [93, 94]. The development of functional nanomaterials has become one of the focuses in the field of biosensors.

Early diagnosis of cancer biomarkers with high sensitivity, low detection limit, and quick analysis plays crucial role in the therapy of cancers. The diagnosis of cancers by assembled peptide-based biosensors has attracted full attention from researchers. Previously, Chen et al. developed an eye-observable detection indicator based on the visual recognition of cancer cells [95]. A biocompatible peptide with a phenylboronic acid moiety (named borono-peptide, BP), B(OH)_2_AEAEAELRARARL-OH, was designed for the detection of cancer cells with overexpressed sialyl Lewis X (sLex). In addition, they developed a flowable catechol dye, alizarin red S (ARS), which had a strong binding ability with phenyl-aldonic acid groups. The color of the solution changed from the free state to the coordination state. It was simply mixed with BP to prepare a visually colored BP/ARS indicator to achieve the goal of visual identification of tumor cells. Therefore, the obtained nano-biosensor had a robust adhesion-induced replacement effect and cancer cells could be in situ observed by the naked eye.

**Fig. 10:**
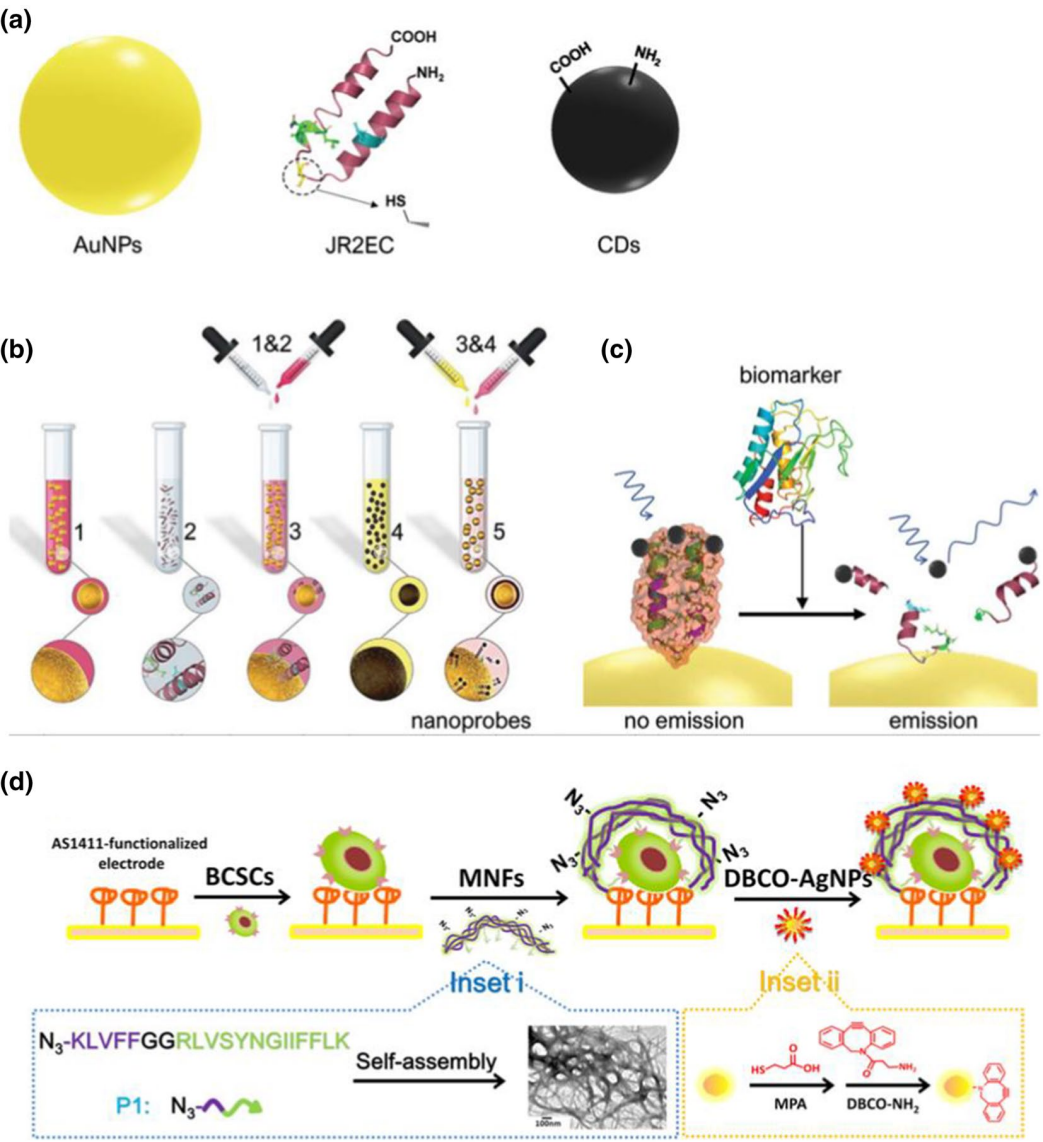
Peptide nano-assemblies for biosensing of cancer cells: **a**–**c** fluorescent detection of cancer biomarker. **a** Components for the synthesis of nanoprobes, **b** step-wise synthesis of nanoprobes, and **c** biosensing mechanism of MMP-7. Reprinted with permission from Ref. [96]., Copyright 2020, Wiley–VCH. **b** Electrochemical detection of cancer cells. Reprinted with permission from Ref. [99], Copyright 2019, American Chemical Society

Recently, peptide nano-assemblies based biosensor platforms, such as fluorescent [96], electrochemical [97–99] biosensors, have been fabricated and used for the detection of cancer cells. For instance, Behi et al*.* reported the fabrication of a fluorescent biosensor based on self-assembled peptide-based nanoprobes for the detection of matrilysin cancer biomarker[96]. As shown in Fig. 10a, the nanoprobe was designed by conjugating peptide JR2EC (NH_2_-NAADLEKAIEALEKHLEAKGPCDAAQLEKQLEQAFEAFE-RAG-COOH) with AuNPs and carbon dots (CDs) together. The JR2EC biopolymer has high affinity to the specific protein biomarkers such as MMP-7. The step-wise synthesis of nanoprobes is presented in Fig. 10b, in which AuNPs were firstly modified with peptide and then CDs were utilized to bind with peptide covalently to form hybrid nanoprobes. After the biomarker (MMP-7) is added into the nanoprobe system, JR2EC is cleaved pyrolytically by MMP-7, resulting the disassembling of nanoprobes and fluorescent increasing due to the releasing of CDs (Fig. 10c). This nanoassembled peptide biosensors exhibited rapid detection (30 s) and low detection limit (30 nM) for the diagnosis of MMP-7.

In addition, electrochemical biosensors based on peptide nano-assemblies for cancer diagnosis have also reported. Yaman and co-workers demonstrated the synthesis of peptide nanoparticles for the fabrication of a sensitive electrochemical detection platform for cytosensing of DLD-1 cancer cells [98]. Diphenylalaninamid (FFA)-based peptide was designed for the synthesis of peptide nanoparticles with a mean size of 400 nm, which were then used to modify pencil graphite electrodes (PGE) for the fabrication of electrochemical biosensors. As the FFA nanoparticle-modified PGE revealed high affinity to DLD-1 cells, low-concentrated DLD-1 cancer cells could be early determined using electrochemical impedance method.

In another case, Tang and co-workers reported the fabrication of electrochemical biosensors based on self-assembled peptide nanofibers for detecting breast cancer stem-like cells (BCSCs) [99]. Figure 10d shows the peptide nano-assemblies based fabrication of biosensor platform and sensing mechanism. Firstly, AS1411 aptamers were used to functionalize the electrode surface, and then BCSCs were added to bind onto the electrode via specific interaction between aptamers and BCSCs. After adding peptide-base multifunctional nanofibers (MNFs), the MNFs could be bound onto stemness marker CD44 through specific interactions. Electrochemically active labels, dibenzocyclooctyne (DBCO)-modified AgNPs were conjugated onto MNFs for creating electrochemical signal. The fabricated biosensors exhibited detection limit of 6 cells/mL with a wide linear range from 10 to 5 × 10^5^ cells/mL.

Beside the fluorescent and electrochemical biosensors, electrochemical immunosensor based on self-assembled peptide nanowires and glucose oxidase has also been fabricated for sensitive detection of tumor necrosis factor α [98]. The electrochemical biosensors of cancer cells have several advantages, such as simple, low-cost, rapid, sensitive, and selective, showing promising perspectives for practical clinic diagnosis of cancers.

## Peptide nano-assemblies for cancer therapy

Peptide-based nano-assemblies have been widely used for cancer therapy due to their uniform nanoscale structure, tailorable functions, high biocompatibility, and specific targeting to cells. In this section, we summarize the peptide assembly techniques for directly killing cancer cells, the role of peptide drug assembly and peptide hybridization in chemotherapy, the application of peptide hybridization in PTT, and the application of peptide hybridization in killing cancer cells with multiple therapies.

### Peptide assemblies for direct killing cancer cells

The difference between cancer cells and normal cells lies in the difference in acquisition function. When the drug kills the cancer cells, it will destroy the normal cells at the same time [100]. The ideal cancer treatment should selectively kill cancer cells without harming normal cells. Current cancer chemotherapies, however, still do not meet this goal. For example, cisplatin (CDDP) [101], a recognized clinical anticancer drug, leads to bone marrow suppression. Another widely used anticancer drug, paclitaxel, has toxic effects on bone marrow. How best to kill cancer cells?

Of the 30,000 genes predicted by the human genome, only about 3,000 genes encode proteins capable of binding small-molecule drugs [102]. The 3, 000 available genes, only 600 to 1, 500 disease-related genes are potential drug targets. Although the number of small-molecule drug targets is limited, peptide assembly can be used to directly target cancer cells [103]. In recent years, techniques for killing cancer cells through peptide assembly have been widely developed. For example, the aggregation of small molecular peptides can selectively inhibit cancer cells, while they are harmless to normal immune cells. Feng and co-workers [104] illustrated the C-terminal modification of short peptides as an effective approach to modulate the self-assembly of the peptides, thus providing a new dimension for exploring enzyme-instructed self-assembly for controlling the fate of the cells. This observation warrants further investigation since it may offer new insights into the use of peptide assemblies for inhibiting cancer cells.

**Fig. 11:**
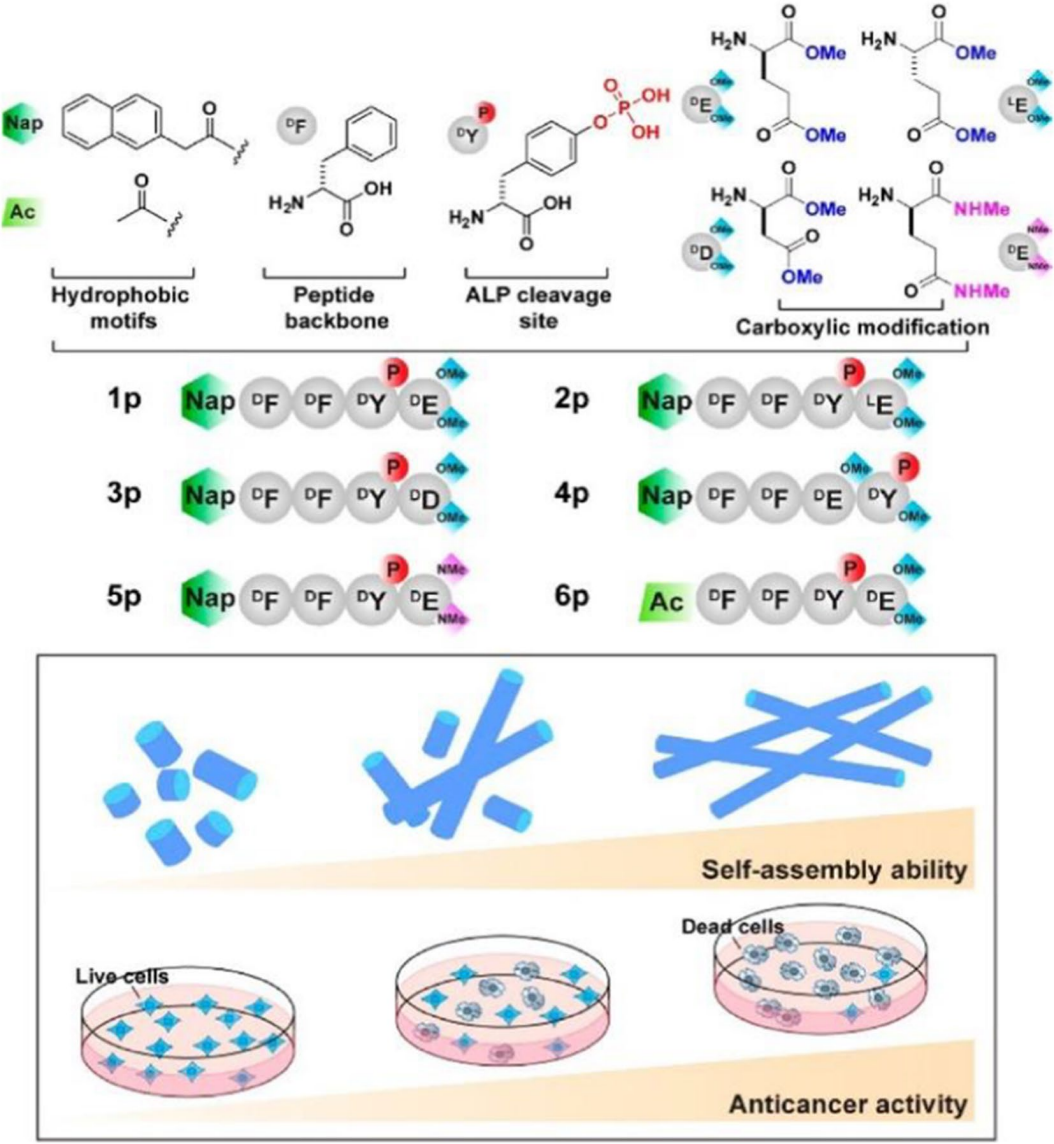
Molecular structures of the precursors and the correlation between the ability for self-assembly of small molecules and anticancer activity. Reprinted with permission from Ref. [105]. Copyright.2017. American Chemical Society

In the process of peptide directly killing cancer cells, the small peptide molecule is expected to selectively inhibit cancer cells, nevertheless, there are still several key problems to be solved. Particularly important is how to design a small molecule for enzyme-instructed self-assembly (EISA). Feng et al. [105] demonstrated in their study that the self-assembly of small molecules controls EISA's anticancer activity. They show that the self-assembling ability of small molecules controls the anticancer activity of EISA. By examining the EISA precursor analogues consisting of an N-capped D-tetrapeptide, a phosphotyrosine residue, and a diester or a diamide group, it was found that regardless of the stereochemistry and the regiochemistry of their tetrapeptidic backbones, the anticancer activities of these precursors largely match their self-assembling abilities. The synthesis scheme of the proposed peptide precursor is shown in Fig. 11. This method illustrates the importance of designing structural analogues of peptide precursors. The results suggest that the self-assembly ability of these peptide derivatives determines EISA's ability to inhibit the anticancer activity of cancer cells. Besides, many other researchers have designed small molecular precursors for controlling the peptide assembly to kill cancer cells. For example, Wang et al. [106] validated the concept of using the molecular process for multi-targeting by rationally designing the precursors consisting of a peptide segment of EISA and a mitochondria-targeting motif. In another case, Zhang et al. [107] designed a responsive small molecule peptide precursor, meanwhile, the precursor was self-assembled into sturdy pages at the tumor site, showing the assembly-induced retention (AIR) effect, adopting the photoacoustic (PA) imaging signal. Their study is thought to be effective in improving cancer treatment.

In addition, there are other ways to use peptide assembly were explored for killing cancer cells. For example, Xu et al. [108]*.* reported a new method to regulate the collaboration of peptide tree macromolecules and linear peptides with self-assembling garment shell-like nanostructures. Jeena et al. [109] described a way to kill cancer cells by assembling the supramolecular fibers of the peptide amphiphiles in the mitochondria of living cells. The program assembled peptides destroy the membrane integrity of the mitochondria, causing severe damage to the mitochondria and leading to leakage of mitochondrial contents, including proteins, into the cytoplasm, ultimately leading to apoptosis. This approach is considered to have great medical prospects and provides new opportunities for further study of organ-specific supramolecular systems for treatment and cellular function.

For biomedical applications, many biologically responsive peptide nanosystems such as pH-responsive and tumor microenvironment controlled peptide nanosystem assembly have been developed. An endowment of pH responsivity to anticancer peptides is a promising approach to achieve better selectivity to cancer tissues. Tanishiki et al. [110] designed a template peptide based on the anti-cancer peptide magainin 2, which has anti-cancer activity. On the template, a series of peptides were designed by replacing different amounts of lysine with unnatural amino acids, 2,3 diaminopropionic acid (Dap), which has a positive charge at weakly acidic pH in cancer tissues but is neutral at physiological pH of 7.4. A peptide with larger cationic charges at a weakly acidic pH than that at neutral pH should exhibit stronger electrostatic interactions with anionic membranes in cancer tissues. Such acidity-responsive tumoricidal (ART) peptides are expected to exert more potent effects against progressed cancers. In another case, Wang et al. [111] developed a nanoscale micelles system (named as HEKM),which consists of tumor microenvironment-regulated shape-changing with specific recognition abilities for enhanced cellular targeting, internalization and therapy of heterogenetic tumors. HEKM can recognize and bind tumor markers, thereby enhancing tumor targeting. In particular, HEKMs can self-assemble into nanorods under normal physiological conditions, and at the same time transform into nanospheres in the microenvironment of tumor cells through a sensitive response to matrix metalloproteinase 2 (MMP-2). The nanorods can extend blood circulation, while the nanospheres can accelerate the penetration of tumor tissue. In vitro and in vivo studies have shown that these reasonably designed and shape-changing targeted micelles can achieve the greatest efficacy and minimal side effects.

To sum up, these conclusions and experimental results indicate that peptide assembly can effectively inhibit the proliferation of cancer cells [112], which provides theoretical and experimental basis for the treatment of cancer and the understanding of the cytotoxicity of pat.

### Peptide-drug assemblies for chemo-therapy

Chemotherapy is a systemic treatment means [113], by oral, intravenous, vascular interventional therapy. Cancers can be cured through chemotherapy. Chemotherapy is known as a prevalent approach for cancer treatment through preventing cancer cell metastasis, Nevertheless, for cancer treatment, chemotherapy has some drawbacks such as limited efficacy, severe toxic side effects, and the tendency to induce drug resistance [114]. To overcome such long-standing challenges, various tumor targeted drug delivery systems have been developed to improve therapeutic efficiency and minimize toxic side effects. For example, Qiao et al. [115] developed a method of peptide drug assemblies for chemo-therapy, namely self-assembly of cytotoxic peptide conjugated poly(B-amino ester)s for synergistic cancer. Ying et al. [116]. Reported a summary approach to prepare filamentous supramolecular peptide-drug conjugates with precise drug carrier stoichiometry, which has nearly 100% loading efficiency and exceptional anti-cancer drug efficacy for chemotherapy. These reports indicate that peptide-drug conjugate has been explored as anticancer drugs and has a wide range of clinical applications.

Targeting peptide-drug delivery is a promising strategy to overcome the side effects of systemic drug-based therapies, including chemotherapy. On the basis of this research, Boekhoven et al. [117] described a system for targeted drug delivery using alginate peptide amphiphilic nucleocapsid particles. Then, they presented the synthesis of nucleic shell particles composed of polymer nuclei and amphiphilic peptide nuclei. The spherical geometry of the particle core allows high drug loading per surface area, and it also illustrates the potential on adopting peptide amphiphiles as effective targeted drugs.

With regard to the amphiphiles, the self-assembly of peptide-drug conjugates into supramolecular nanomaterials has been reported [118]. For example, Lin *et al*. [119] reported the use of catanionic mixing of anticancer drug amphiphiles to construct multiwalled nanotubes containing a fixed and high drug loading. Consequently, in their experiment, the packing requirements of the hydrophobic core within the assemblies formed by catanionic mixtures (CAMs) of qCPT-Sup35 which play a crucial role for the mixtures to adopt tubular structures as their dominant supramolecular morphology. The forming process is likely a cumulative result of 1D elongation, multiple bilayer formation, and bilayer extension. The above experimental results show the great potential of peptide drug self-assembly into discrete nanostructures to kill cancer cells, providing an important basis for the study of the formation mechanism of carbon nanotubes, and supplying a broad research prospect for the extensive use of peptide drug assembly technology in chemotherapy [120].

**Fig. 12:**
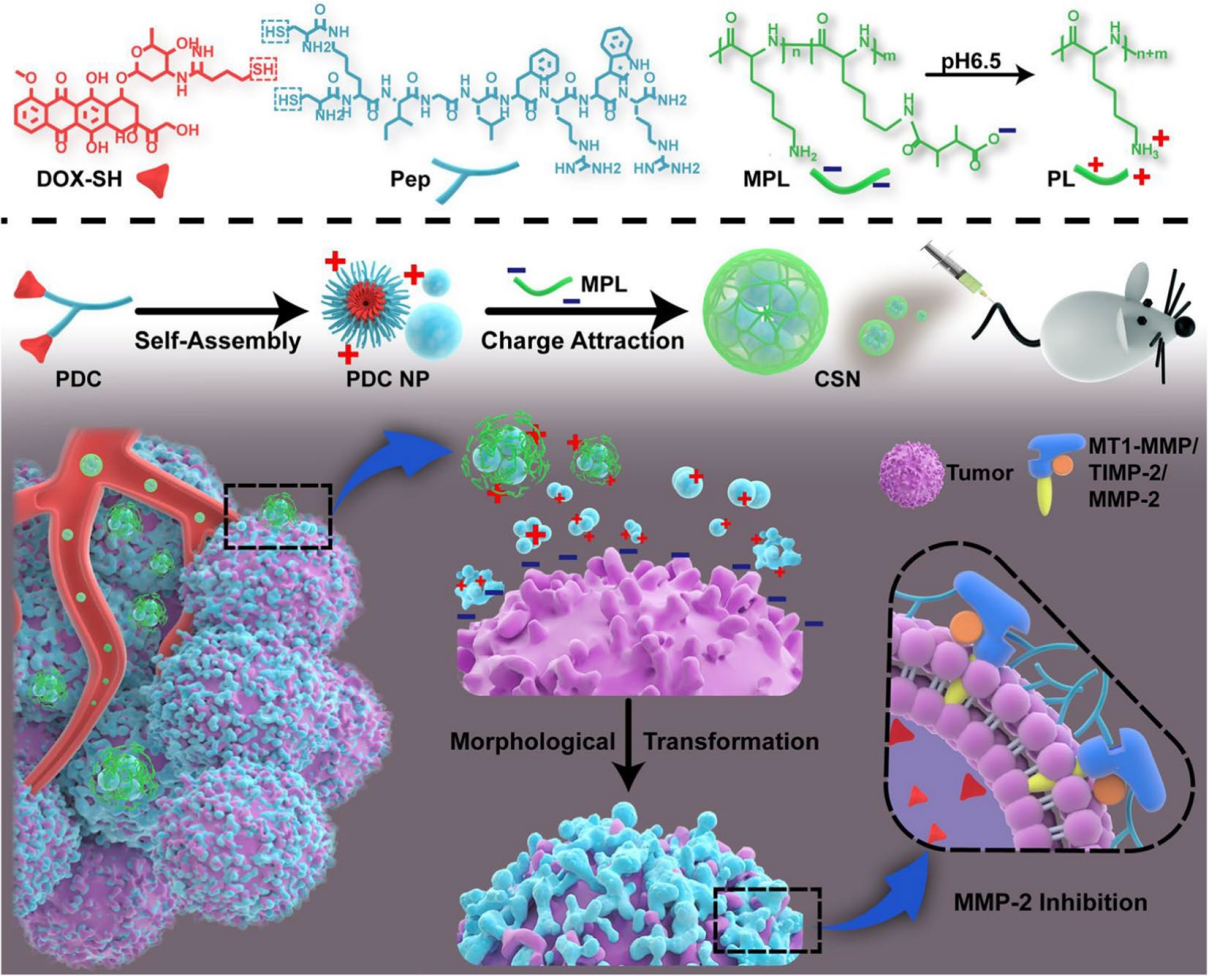
Schematic Illustration of the Construction of CSNs and Construction based on morphological transformation for high-efficient tumor killing and inhibition of tumor metastasis. Reprinted with permission from Ref. [121]. Copyright. 2020. American Chemical Society

Cancer metastasis is the main cause of chemotherapeutic failure. Inhibiting the activity of matrix metalloproteinases (MMPs) is a common strategy for reducing metastasis. However, broad-spectrum MMP-inhibitors (MMPI) may cause undesired side effects. Qian *et al*. [121] screened a selective MMP2 inhibitor (CCKIGLFRWR) and linked it with DOX to produce an amphiphilic peptide-drug conjugate (PDC) (Figure 12). The disulfide bond between the MMPI peptide and DOX was broken via a low concentration of glutathione-mediated reduction in tumor microenvironment. Experiments have shown that CSNs are more efficient than DOX or PDC in inhibiting tumor growth and preventing tumor metastasis, achieving a "siege" effect. In addition, MMPI peptide and DOX are covalently linked to each other, avoiding early drug leakage and reducing the side effects of other tissues.

It is necessary to deliver a drug with a prescribed molecular state in order to maximize the efficacy of peptide-drugs in chemotherapy [122]. For example, Lu et al. [122] investigated the molecular binding of self-assembled peptide EAK16-II to the anticancer drug Elliptic peptide (EPT), and illustrated the significance in cancer cell inhibition. They presented a simple method to characterize the molecular states of the anticancer drug EPT encapsulated in self-assembled peptide EAK and to correlate their different molecular states with their respective anticancer efficacy. Moreover, the potential application of peptide-drug assembly in chemotherapy was also demonstrated.

### Peptide-based hybrid assemblies for chemo-therapy

The application of peptide-based hybrid components in chemotherapy has great advantages as a therapeutic approach for tumor therapy, as they can effectively avoid the annoying problems such as low efficiency and side effects of anti-tumor drugs. Recently, a list of published works have reported the progress of the relevant research. For example, Peng et al. [123] reported a peptide hybridization for chemotherapy and the approach is self-delivery of peptide-based prodrugs for tumor targeted chemotherapy. In their approach, peptide-based hybridization for tumor therapy can effectively reduce the adverse reactions such as poor cellular uptake and low fibrillation effect. They also provided an idea for the design of a new carrier-free system for tumor targeted therapy.

The technology of peptide-based hybrid assembly continues to attract attention among researchers due to their potential applications in various fields. Peptide molecules are not only good gelatos due to the presence of various self-assembling units in their structures, but also possess inherent biocompatibility which enhance their applicability. Interestingly, gel stiffness, drug release capacity and proteolytic stability of these hydrogels have been successfully modulated by incorporating D-amino acid residues, indicating their potential application for drug delivery [124]. Moreover, all peptide based gelator molecules are chiral gelator molecules, and whose chirality can be a tool for tuning the physical properties of the corresponding gels to make them smarter. The simplicity and low cost of synthesis make these peptide-based materials a wonderful platform for chemotherapy.

Chemotherapy is still an effective treatment for cancer [125], and peptide-based hybridization had a profound impact on cancer treatment in the past decade. In another case, for example, Xiao et al. [126] reported the development of the redox-responsive mesoporous silica nanoparticle (RRMSN) as a drug nanocarrier for the delivery of cancer-targeted drugs that are peptide-hybridized and have a redox reaction with amphiphilic peptides. The proposed novel RRMSN/DOX drug delivery system was self-assembled by amphiphilic peptide based on MSNs. This novel RRMSN/DOX drug delivery system developed by self-assembly of amphiphilic peptides onto MSNs would provide a facile, but effective strategy for the development of smart and targeted drug carriers for cancer therapy.

These studies have proved that peptide-based hybrid assembly has the advantages of quick effect and low toxicity in chemotherapy, and has a broad application prospect in cancer treatment [127].

**Fig. 13:**
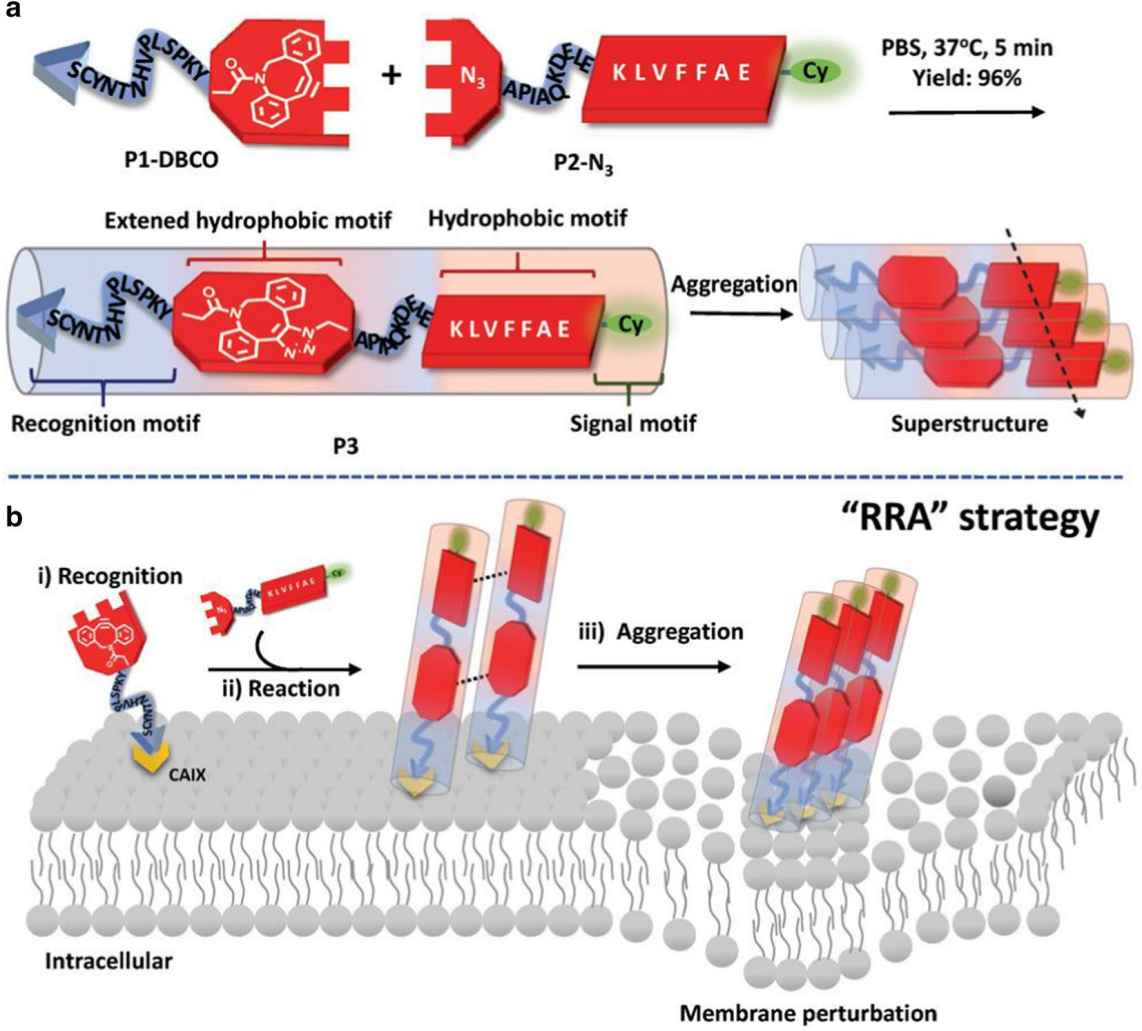
Schematic illustration of the molecular structure and the RRA cascade process. **a** The modular structure of DBCO-linked CAIX-targeting peptide (P1-DBCO) and azido-linked peptide labeled with cyanine dye (P2-N3). **b** At the cellular level, P1-DBCO can specifically recognize renal cancer cells by targeting CAIX.Then P2-N3 is added to react with P1-DBCO to form monomeric peptide P3 on the cell membrane.At the same time, the monomer P3 will gather to form a superstructure to achieve membrane disturbance. Reprinted with permission from Ref. [128], Copyright 2019, Wiley–VCH

Chemotherapy has been validated unavailable for treatment of renal cell carcinoma (RCC) in clinic due to its intrinsic drug resistance. However, in recent work, a breakthrough has been made on this issue. Wang *et al*.[128] first in situ construct the self-assembled superstructure on the cancer cell membrane through recognition–reaction–aggregation (RRA) strategy, enabling the enhancement of chemodrug sensitivity of renal cell carcinoma. The general design scheme of the structure and the RRA strategy process on the cell membrane are shown in Figure 13. Genally, the RRA strategy is a cascading process, including three steps. i) Recognition: P1-dibenzocyclooctyne (DBCO) specifically recognized renal cancer cells by targeting carbonic anhydrase IX (CAIX) which was abundantly expressed in RCC and almost no expression in healthy tissues. ii) Reaction: P2-N3 was consequently introduced and reacted with P1-DBCO by reagent-free click chemistryto form an aggregable peptide P3. iii) Aggregation: the synthesized P3 simultaneously aggregated into superstructures owing to the extended hydrophobic unit and unbalanced hydrophilic–hydrophobic interactions. Interestingly, the superstructure remains on the cell membrane and disrupts its integrity/permeability, allowing kidney cancer cells to absorb more DOX [128]. Finally, the RRA strategy significantly inhibited tumor growth in xenograft mice, and the inhibition rate increased by 3.2 times. This newly developed RRA strategy will open up a new way for chemically engineered cell membranes with multiple biomedical applications.

### Peptide-based hybrid assemblies for PTT

Photothermal therapy (PTT) is a promise and elegant method for tumor treatment, which has the advantages of minimum invasiveness, easy procedure, short treatment time, and quick recovery [129]. PTT relies heavily on the use of photothermic agents, which are essential for the effective conversion of light energy into heat for the ablation of cancer cells and tumors[130]. Photothermal agent is also a new contrast agent for photoacoustic imaging diagnosis technology, which has deeper penetration and higher resolution in biological tissues than traditional optical imaging technology. Compared with conventional resections, peptide-based hybrid assemblies for PTT have numerous beneficial such as minimal invasion, relatively simple execution, and particular direct heating in the tumor [130], which exhibit huge application potential for cancer treatment.

Recently, Zou et al. [131] achieved the fabrication of photothermal nanodots of around 25 nm diameter with long-term colloidal stability by tuning the molecular interactions of the peptide porphyrin conjugates. The transmission electron microscopy (TEM) images disclose that the assembled peptide-porphyrin photothermal nanodots (PPP-NDs) are in a regular spherical shape with the size of around 28 nm, almost consistent with the dynamic light scattering (DLS) result. Subsequently, they monitored the photochemical transformation under irradiation with an infrared thermography camera and observed the average temperature of the tumor site. Moreover, the tumor volume of mice in each group was also monitored, and no obvious pathological changes or other adverse reactions were noted.

In recent years, peptide-based hydrogels represent attractive molecular building blocks due to their ease of synthesis, versatile gelation approaches, and excellent biocompatibility. For the delivery of anticancer drugs, especially in PTT, it would be desirable to load photothermal conversion agents (PTCAs) together with therapeutic agents to produce synergistic antitumor effects for reducing the tumor recurrence rate. For example, Jin et al.[132] used melittin-containing peptide-based hydrogels to enhance photo thermotherapy in glioblastomas. The novel melittin RADA32 indocyanine green (ICG) hydrogel (MRI hydrogel) contains melittin in the peptide hydrogel backbone and ICG in the hydrogel matrix which enhance the photothermal therapy of glioblastomas.

**Fig. 14:**
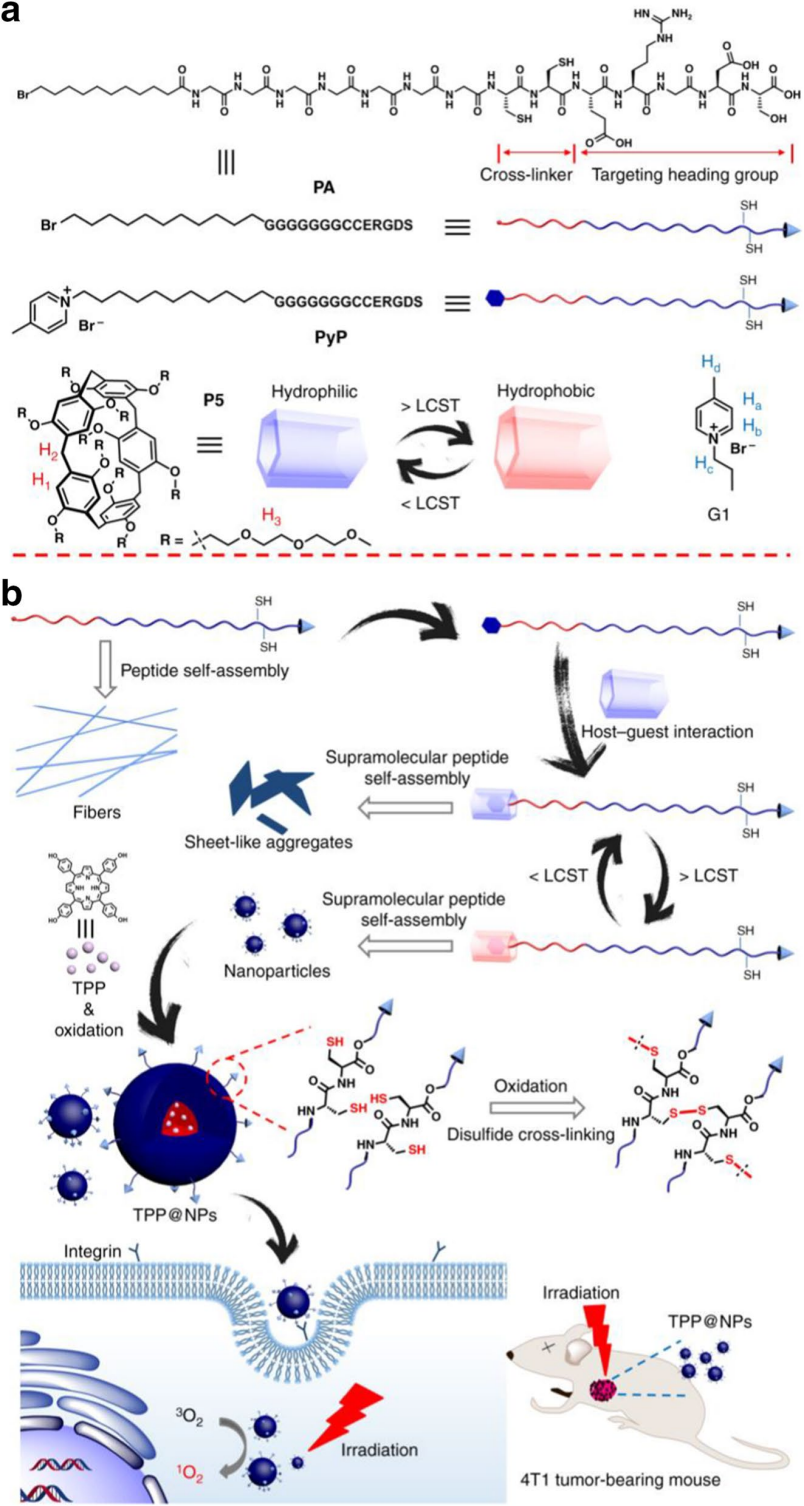
Schematic diagram of the design and construction of supramolecular peptides. **a** Chemical structure and cartoon representation of PA, PyP and P5. **b** Schematic diagram of programmable peptide self-assembly and PDT process. The solid arrow indicates the experimental procedure, and the hollow arrow indicates the self-assembly or structural details. Reprinted with permission from Ref. [133], Copyright 2019, Nature Publishing Group

The self-assembly of peptides with a variety of nanostructures has great potential in functional biomaterials [134]. However, poor morphological controllability of covalent peptide self-assembly and tedious covalent modification of peptides hinder the exploration of peptides. Zhu et al. [133] reported a simple method for making supramolecular peptides, which showed programmable self-assembly with multiple morphologies and potential applications in photodynamic therapy. The peptide sequence (see Fig. 14a, G7CCERGDS, G = glycine, C = cysteine, E = glutamic acid, R = arginine, D = aspartic acid, S = serine) was designed as follows: (1) Multiple hydrogen-bonded tight hydrophobic cores play an indispensable role in self-assembly to form nanostructures, so seven glycine moieties are used to form strong multiple hydrogen bonds; (2) Cysteine units are easily oxidated by forming a disulfide cross-linking agent. (3).The ERGDS heading group promotes the cellular internalization of the self-assembled NPs by cancer cells overexpressing αvβ3 integrin through receptor-mediated endocytosis. The self-assembled morphology of this supramolecular peptide, including sheets and nanoparticles, can be easily controlled by adding P5 and heating, instead of cumbersome covalent modification of the peptide (Fig. 14b). In addition, the cysteine unit on PyP is oxidized into a disulfide cross-linked shell, which prevents the collapse of NP after the temperature is lowered. Disulfide bonds also show a response to glutathione (GSH) and may consume intracellular GSH to synergistically increase the efficiency of photodynamic therapy (PDT). Due to the outer ERGDS sequence and self-assembly of hydrophobic cores, supramolecular peptide nanoparticles are encapsulated photosensitizer treatment with light power suitable carrier. In vitro and in vivo studies have shown that its inherent targeting ability and supramolecular strategy greatly improved the efficiency of photodynamic therapy.

### Peptide-based hybrid assemblies for multi-therapy

Cancer therapy via single means such as chemotherapy has certain limitations including restricted curative effect, serious toxic side effects and easy drug resistance. To overcome these long-standing drawbacks, a variety of tumor-targeted drug delivery systems has been developed towards treatment efficiency improvement and side effects minimization. On the other hand, the combination of multi-therapy strategies is another promising strategy to improve the efficiency of treatment and overcome drug resistance. Various therapies such as chemotherapy, radiation therapy, gene therapy, magneto thermic therapy, photothermal therapy and photodynamic therapy can be combined to achieve a synergistic anti-tumor effect. In recent years, the combination of photothermal therapy and chemotherapy has been explored for tumor treatment. For instance, Huang et al. [135] successfully designed a novel targeted drug delivery system based on functionalized gold nanocages (AuNCs). The results showed that the intracellular enzyme hyaluronidases (Hyal) could trigger doxorubicin (DOX) to release, while NIR laser and acidic pH could accelerate this process.

The peptide-based hybrid assemblies for multi-therapy plays an integral role in most cancer treatments, as it can overcome the problems of multi-drug resistance and tumor recurrence and metastasis. In a recent study, Wu et al.[136] prepared a plasma-lactose coated antitumor drug double-loaded polypeptide/gold nanoparticles under mild conditions for the first time. The integration of photothermal therapy and two anticancer drug-induced cocktail of chemotherapy into a biodegradable and biocompatible polymer nanocarrier shows a synergistic cocktail of photothermal chemotherapy for cancer. These experiments demonstrate the great advantages of multi-therapy based on peptide assembly. Moreover, multi-therapy shows a good synergistic anti-tumor effect. It was also confirmed that the combination cocktail chemo-photothermal therapy produced a lower half maximal inhibitory concentration than cocktail chemotherapy or photothermal therapy alone, displaying a good synergistic antitumor effect. In another case, Thapa and co-workers [137] studied the hydrophobic binding peptide-conjugated hybrid lipid-mesoporous silica nanoparticles, which can be effective chemo-photothermal therapy of pancreatic cancer. The combination of chemotherapy and phototherapy therapy is a potential viable approach for the treatment of pancreatic cancer, as it would treat the tumor by involving different synergistic pathways (DNA damage, ROS generation and photothermal ablation), leading to cancer cell apoptosis.

**Fig. 15:**
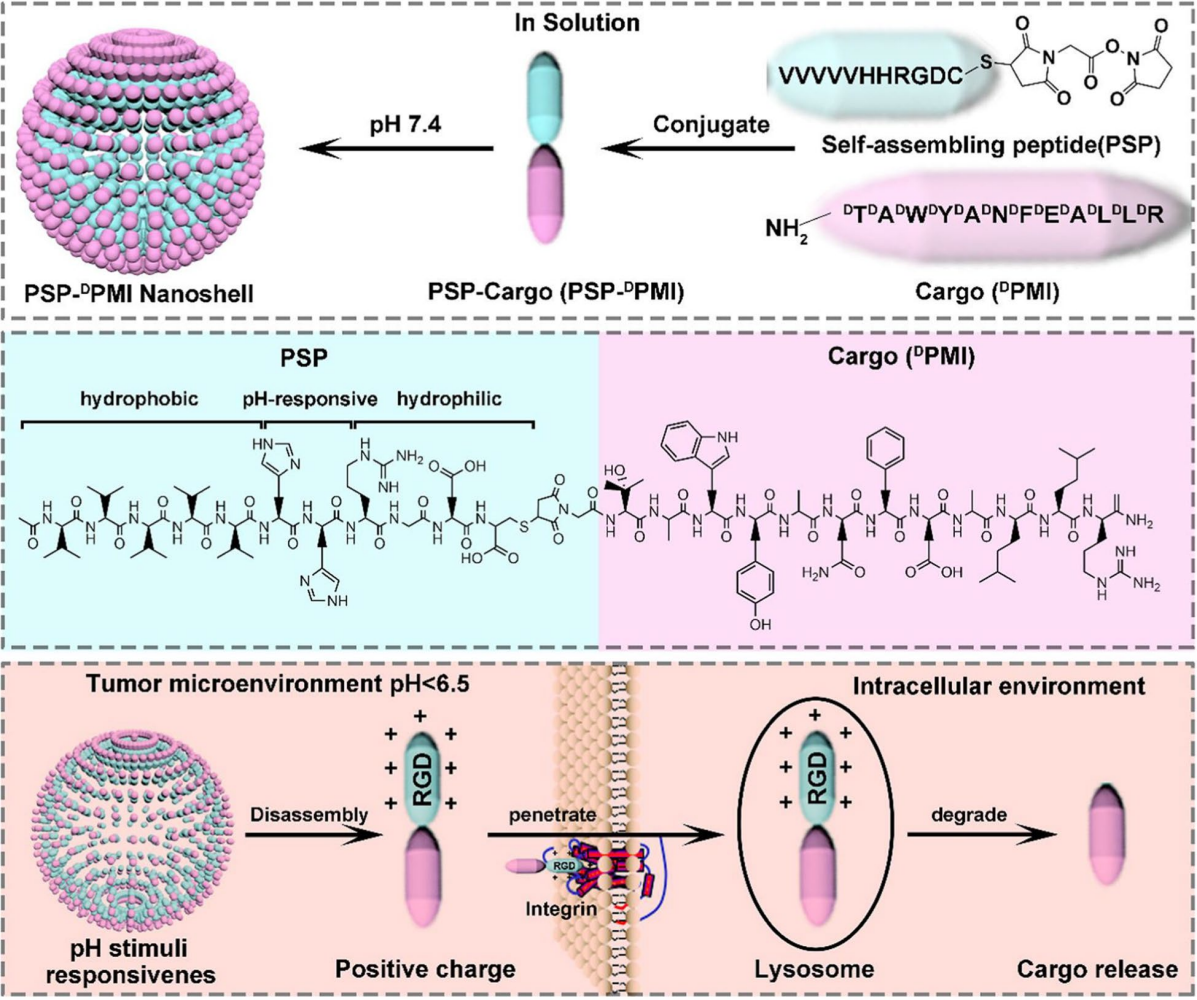
Schematic illustration of self-assembly synthetic procedure and the properties of pH-responsive charge reversal and size change of PSP-DPMI spherical shells. Reprinted with permission from Ref. [138], Copyright 2018, American Chemical Society

At present, the drug delivery of peptide self-assembled nanoparticles is still hindered by poor tumor specificity and short circulation time, resulting in poor therapeutic effects. The ideal delivery system should be relatively large and negatively charged in its initial state to achieve a longer circulatory half-life and selective extravasation. It can also be switched to positively charged small particles according to the tumor microenvironment (TME) response of the tumor site to promote tumors infiltrate. Multi-therapy is a promising way to give these nanoparticles a tumor microenvironment (TME) response, which can usually mediate the specific formation or fracture of certain nanostructures. He et al. [138] developed a new strategy for nanoengineering macromolecular drugs by an elaborate peptide, termed as PSP (VVVVVHHRGDC), which is capable of directly conjugating with cargo to be a PSP-cargo monomer as building block tending to self-assemble into a well-defined nanoshell with tumor-triggered shape and charge switch (Fig. 15). As a proof of concept, conjugation PSP to a D-peptide activator of tumor suppressor p53 termed ^D^PMI (1492.5 Da) generated hollow spheres ∼80 nm in diameter named as PSP-^D^PMI that disintegrated only in the acidic microenvironment of tumor tissues, followed by integrin-mediated cellular uptake of PSP-^D^PMI monomers. Taking full advantage of the EPR effect, tumor microenvironment sensitivity and RGD targeting, PSP-^D^PMI can specifically accumulate at the tumor site with a long residence time. In vitro and in vivo experiments show that PSP-^D^PMI has strong anti-tumor activity and excellent biological safety. This overall strategy will simultaneously overcome the technical barriers of self-assembled nanoparticles as macromolecular drug carriers, as well as the drug barriers of using peptides to regulate the self-assembly of macromolecular drugs into TME-triggered macromolecular therapies in clinical settings [138].

With the development of multi-therapy technology, the integration of peptide-based hybrid assemblies [139] into various therapeutic technologies has become a major trend. Recently, peptide-based hybrid assemblies for multi-therapy also have great significance for the treatment and diagnosis of Alzheimer's disease (AD). A novel strategy was presented by self-assembly of peptide conjugated Au nanorods (AuP) as multifunctional Aβ fibrillization detectors and inhibitors [140]. Compared with traditional drug strategies, the functional AuP possesses the potential ability to cross the blood–brain barrier (BBB) which overcomes the drawbacks of unstable β-sheet breaker peptides. Moreover, this study provides new insights into the design of new multifunctional nanomaterial for both sensitive detection of AD pathology and effective treatment of AD.

Besides, many drug-delivery systems (DDSs) based on various functional nanoparticles have been widely explored for applications in cancer multi-therapy. Chen et al. [114] further reported a simple method to construct albumin-based theragnostic nanoplatforms via drug-induced protein assembly to realize tumor-targeted cancer treatment with combined photodynamic/chemotherapy. This new type of tumor-targeted multifunctional albumin-based nanoparticles by drug-induced self-assembly is a facile method without any sophisticated chemistry or materials engineering, which is promising for multimodel imaging-guided multi-therapy of cancer.

## Conclusions and outlooks

In summary, we presented the progress on the design and synthesis of functional peptide nano-assemblies for cancer diagnosis and therapy applications. Multifunctional peptide nano-assemblies exhibited wide applications for the diagnosis of cancers, such as fluorescent and magnetic imaging as well as biosensing of cancer cell biomarkers. Meanwhile, peptide nano-assemblies could conjugate with other components such as metal nanoparticles, QDs, drugs, photosensitive polymers and others to enhance their applications in cancer therapy through biological, chemical, photothermal, magnetic, and combined therapy methods. Compared to traditional nanomaterials for cancer therapy, peptide nano-assemblies revealed a few key advantages. They showed very high biocompatibility, bioactivity, and biodegradability, which are useful for biomedical studies. In addition, they provided adjustable structures from 0 to 3D, which exhibited structure-specific applications. For instance, peptide nanoparticles, nanospheres, and vesicles are beneficial for the loading and releasing anticancer drugs; peptide nanofibers and nanosheets showed importance for photothermal and photodynamic therapies of cancers. Besides, they provided the infinity of space to tailor the functions of synthesized materials. Specific peptide motifs can be designed to improve the functions of binding, targeting, cell-penetration, biomimetic synthesis, and others, promoting the function-specific applications of peptide nano-assemblies by choosing appropriate therapy methods and techniques.

Although great achievements have been previously reached on the design, synthesis, and cancer diagnosis/therapy applications of peptide nano-assemblies, here we would like to suggest several potential topics in this promising research field to expand the scope and development of peptide-based nano-assemblies. To start, the motif-design technique is recommended to study the self-assembly of peptide functional conjugates for the formation of novel peptide nano-assemblies with tumor cell-specific properties. The rational design of tumor cell-specific peptide sequences and adjusting the self-assembly conditions in vitro and in vivo could beneficial for this topic. Second, it is necessary to investigate deeper the self-assembly of peptides with designed motifs in live cells, for instance the assembly and disassembly of peptide nano-assemblies under the catalysis of cancer-relevant enzymes and cancer biomarkers. Both experimental and theoretical studies will promote the development of biological agents for in-situ cancer diagnosis and therapy of cancers. Third, as 2D biological materials exhibited higher specific area and more active sites, 2D peptide nano-assemblies are highly interesting for the synthesis of functional 2D materials for imaging, detection, and therapy of cancers. Therefore, peptides could be sequence-tailored to form 2D peptide nanosheets, nanobelts, and nanoplates, which could be utilized as versatile platforms for advanced applications. Fourth, to improve the functional tailoring of peptide nano-assemblies, it is possible to conjugate peptide nano-assemblies with functional 2D materials such as MXene nanosheets, black phosphorus nanosheets, metal–organic frameworks to enhance the cancer diagnosis and therapy efficiency. Finally, more attention should be focused on the development of practical products based on peptide nano-assemblies for cancer diagnosis and therapy. These products should have the characteristics of low cost, high bioactivity, good performance. For instance, it is possible to develop peptide nano-assemblies based rapid detection nanosensors, test kits, peptide nanodrugs, and imaging contrast agents. We believe that supramolecular peptide nano-chemistry will promote the connection between nanotechnology and biotechnology, and show more positive effects on solving problems in disease diagnosis and treatment in the future.
